# D-amino acid metabolic versatility as a common adaptive strategy in the Mariana Trench microbiome

**DOI:** 10.1128/msystems.00581-25

**Published:** 2025-07-11

**Authors:** Xiangyu Wang, Yongxin Lv, Weishu Zhao, Xiang Xiao, Jing Wang

**Affiliations:** 1 State Key Laboratory of Microbial Metabolism, School of Life Sciences and Biotechnology, Shanghai Jiao Tong University 553742 , Shanghai, China; 2 International Center for Deep Life Investigation (IC-DLI), Shanghai Jiao Tong University 12474, Shanghai, China; 3 School of Oceanography, Shanghai Jiao Tong University 722366, Shanghai, China; 4 Yazhou Bay Institute of Deepsea Sci-Tech, Shanghai Jiao Tong University, Sanya, China; Monash University, Melbourne, Victoria, Australia

**Keywords:** Mariana Trench, D-amino acids, metagenome, recalcitrant dissolved organic matter

## Abstract

Hadal trenches, the Earth’s deepest marine environments, harbor thriving microbial communities that promote the turnover of recalcitrant dissolved organic matter (RDOM) under extreme conditions. However, the effects of microbes on D-amino acid (D-AA) reservoirs, which are important components of deep-sea RDOM, remain largely unknown. To address this knowledge gap, we curated a comprehensive reference database of D-AA functional genes for accurate identification of D-AA metabolic potential from metagenomic data. Using this database, we identified the presence of various D-AA anabolic and catabolic genes that were closely correlated with central carbon metabolism and ammonia oxidation genes throughout the water column and in the sediment of the Mariana Trench. Furthermore, 93.6% of the recovered bacterial and archaeal genomes contained at least one of these D-AA functional genes, substantially expanding our understanding of potential D-AA utilizers. Notably, we discovered that glutamate racemase, an enzyme previously thought to be exclusive to bacteria, is ubiquitously present in ammonia-oxidizing archaea. This finding suggests that D-glutamate could be integrated into hadal carbon and nitrogen cycling by this crucial microbial taxon. Finally, we observed an increase in both D-AA production and degradation potential with water depth, with higher levels in near-bottom seawater than in sediment. These findings suggest that diverse microbial taxa promote increased D-AA turnover in hadal zones, potentially representing a common adaptive response to extreme hadal conditions.

**IMPORTANCE:**

Deep-sea microorganisms play a crucial role in the turnover of RDOM. In this study, we investigated the metabolic potential of D-AAs, which are important constituents of RDOM and are used for indicating the recalcitrance of organic matter. By elucidating the genetic profiles of D-AA metabolism and associated microbial taxa, we observed that D-AA metabolism is a fundamental ecological function that is prevalent in the deepest ocean. Our finding of higher D-AA turnover potentials in deeper environments challenges the conventional view of the constant recalcitrance of D-AAs, suggesting that D-AA turnover may be environmentally dependent. This insight provides a new paradigm for understanding RDOM turnover, with broad implications for marine biogeochemistry.

## INTRODUCTION

Hadal trenches are unique marine environments formed by the subduction of oceanic plates beneath continental plates within regions with water depths between 6,000 and 11,000 m and known as the hadal zone (1). The hadal zone represents one of the most extreme and unexplored ecosystems on Earth, characterized by extremely high hydrostatic pressure (up to 115 MPa) and low temperature (approximately 2°C) (2, 3). Due to the great distance from the euphotic zone, organic matter reaching hadal environments typically has undergone extensive degradation, resulting in aged and recalcitrant compounds, as evidenced by old radiocarbon ages (4–6). In addition, special topographies and frequent tectonic activities cause fluctuations in organic matter input (7), posing further challenges to microbial life. These environmental challenges shape the survival strategies of hadal microorganisms, with the ability to utilize recalcitrant organic matter considered as a key adaptation mechanism. For instance, one of the dominant taxa, Chloroflexi, possesses diverse related metabolic pathways, enabling them to cope with the dynamic “feast-or-famine” conditions in the hadal biosphere (8). Moreover, the aromatic compound utilization has been identified as a key function driven by hadal environmental stress (9).

D-amino acids (D-AAs) have been identified as important components of recalcitrant dissolved organic matter (RDOM), the largest organic carbon reservoir in the ocean (10). The quantification of D-AAs, expressed as the D/L-AA ratio, has been used as an indicator of organic matter recalcitrance (11) and a proxy for assessing the turnover of biomass and necromass (12), making the metabolism and turnover of D-AAs in marine environments a subject of considerable interest (13–15). In particular, higher D/L-AA ratios have been observed in deep water (>1,000 m) than in surface water (16, 17), further underscoring the importance of investigating D-AA cycling in the deepest marine environment. However, few studies have explored whether D-AA utilization is a critical factor in microbial adaptation to hadal environments, and no previous studies have investigated D-AA cycling within these extreme ecosystems.

Recent advances in the identification of D-AA metabolic pathways have provided insights into the diverse physiological functions of D-AAs and enabled the investigation of their production and degradation processes using omics methods. The production of D-AAs is primarily mediated through racemases (18) and transaminases (19). D-AAs contribute to peptide modifications that increase resistance to proteases or antibiotics and play an essential role in bacterial peptidoglycan (mainly D-Ala and D-Glu) (20, 21) and non-ribosomal peptides (NRPs) (22, 23). Additionally, non-canonical D-AAs (NCDAAs) could be produced in response to stress conditions (24, 25). These diverse physiological functions suggest that not only D-AA degradation but also D-AA synthesis may play a role in the adaptation of hadal microorganisms, contributing to processes such as maintaining cell wall integrity and responding to environmental stress.

D-AAs can be released into ecosystems through direct secretion by bacteria (24) or via virus lysis (26), serving as potential carbon and nitrogen sources for microbial communities. The ability to utilize these recalcitrant compounds has been identified in some microorganisms and involves a highly diverse set of enzymes (14, 27, 28). Among these, oxidases/dehydrogenases, lyases, and transaminases can directly catalyze D-AA degradation, whereas direct and indirect racemization pathways convert D-AAs into L-AAs before further breakdown. Notably, deep-sea microorganisms may possess a greater capacity for D-AA utilization than upper-ocean microorganisms do, as evidenced by the increased absorption ratios of D/L-AAs with depth (29) and the finding that D-AA utilization is less impaired under high pressure than L-AA utilization is (30). These observations further support the idea that D-AAs may serve as important nutrient sources in hadal environments.

In this study, we performed a metagenome-based study to investigate the microbial potential for D-AA metabolism in the hadal zone, with 78 metagenomic data sets of Mariana Trench seawater and sediment samples covering a broad range of water depths from 0 to 10,908 m. By integrating the D-AA metabolic pathways reported previously as the basis for annotation of D-AA functional genes, we provide a relatively comprehensive estimation of the microbial contribution to D-AA anabolism and catabolism in the Mariana Trench.

## RESULTS

### Enhanced annotation of microbial D-AA functional genes in the Mariana Trench

To comprehensively annotate the D-AA functional genes, we constructed a custom data set comprising 25 relevant Kyoto Encyclopedia of Genes and Genomes (KEGG) ortholog (KO) identifiers (KO-25, Table S1) and 151 experimentally validated protein sequences (Seq-151, Table S2) from previous studies. Seq-151 was categorized into three groups on the basis of functional characteristics and KO assignment. Group I (*n* = 105) comprised enzymes with D-AA-related functions with annotations that accurately reflected their functionality. Group II (*n* = 41) consisted of enzymes that demonstrated D-AA-related functions but were annotated for different functions; we identified additional potential D-AA functional genes by mining sequences with high identity to these enzymes from their assigned KOs. Group III (*n* = 5) included enzymes that were homologous to Group I/Group II but were experimentally verified as non-functional; these sequences served as valuable references to eliminate false positives in our annotation process.

Phylogenetic analysis of Seq-151 and its homologs within the same KO categories supported our annotation strategy (Fig S1). In general, Group I showed a broad distribution among homologs, reinforcing the reliability of KO-based annotation, whereas Group II formed distinct clades, indicating that only sequences with high identity to Seq-151 should be considered potential D-AA functional genes. The close phylogenetic relationship between Group III and functional enzymes highlights the importance of using these non-functional sequences as negative references during annotation.

**Fig 1:**
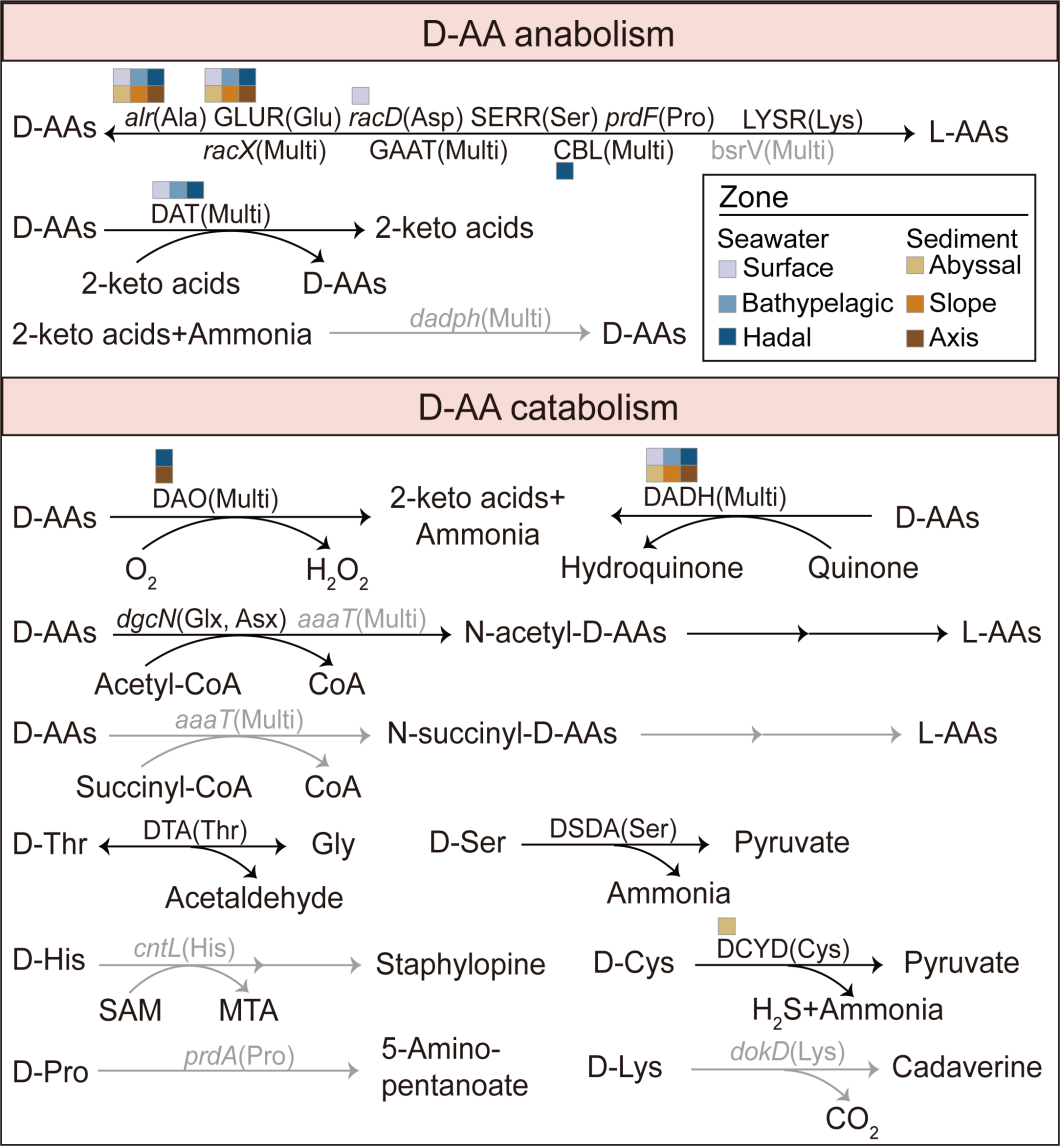
Schematic diagram of D-AA metabolic pathways and related functional clusters. Clusters with mean relative abundances exceeding 0.01% (transcripts per million reads >100) in specific habitats are marked with corresponding squares. The top 16 clusters are marked with black font. The D-AA substrates of the enzymes are annotated in parentheses. Asx, Asp and Asn; Glx, Glu and Gln; MTA, 5′-methylthioadenosine; Multi, multiple D-AAs; SAM, S-adenosyl-L-methionine.

Metagenomic data of the Mariana Trench were retrieved from a public database (*n* = 74) (31–35) or generated in this study (*n* = 4). Specifically, the metagenomes of seawater samples (*n* = 24) collected from depths ranging between 0 and 10,905 m and sediment samples (*n* = 54) from 21 stations at depths between 5,333 and 10,908 m were analyzed (see Table S4 for details of the metagenome data sets). Upon analysis, 39 out of 42 D-AA-related KOs were identified across the 78 samples. A majority of the KOs were shared across the samples, with 29 KOs found in more than half of the samples. On the basis of function or sequence homology, we further clustered the 42 D-AA-related KOs into 23 functional clusters (Fig. 1 and Table S1; see Materials and Methods). A total of 21 functional clusters were identified in the metagenomes; 16 were shared among more than half of the samples (defined as the top 16 clusters). The stable composition of D-AA functional clusters reflected by their relative abundance further supported shared D-AA metabolic function across different samples (Fig S2). The above results indicate the presence of diverse D-AA functional genes in the Mariana Trench metagenomes and that similar D-AA metabolic processes are shared in samples with high geographical isolation.

Among the total annotated D-AA functional genes, the genes that were annotated supplementally on the basis of the alignment to Seq-151 accounted for 25.1% of the total relative abundance of genes (Fig S3). Notably, although both D-Ser and D-Asp are important D-AAs in the marine environment (36), the relative abundance of the serine racemase SERR annotated via KO-25 was only 10.4% of that of the Asp racemase *racD*. However, Seq-151 included two SERR sequences that were misannotated as Ser dehydratases on the basis of KO assignment, the homologs of which represented 90.6% of the total relative abundance of SERR, making the relative abundance of SERR comparable to that of *racD*. These findings indicate that supplemented annotations based on Seq-151 provide a more comprehensive understanding of the microbial potential of D-AA metabolism in the Mariana Trench.

### Diversity of D-AA functional genes among different Mariana Trench habitats

**Fig 2:**
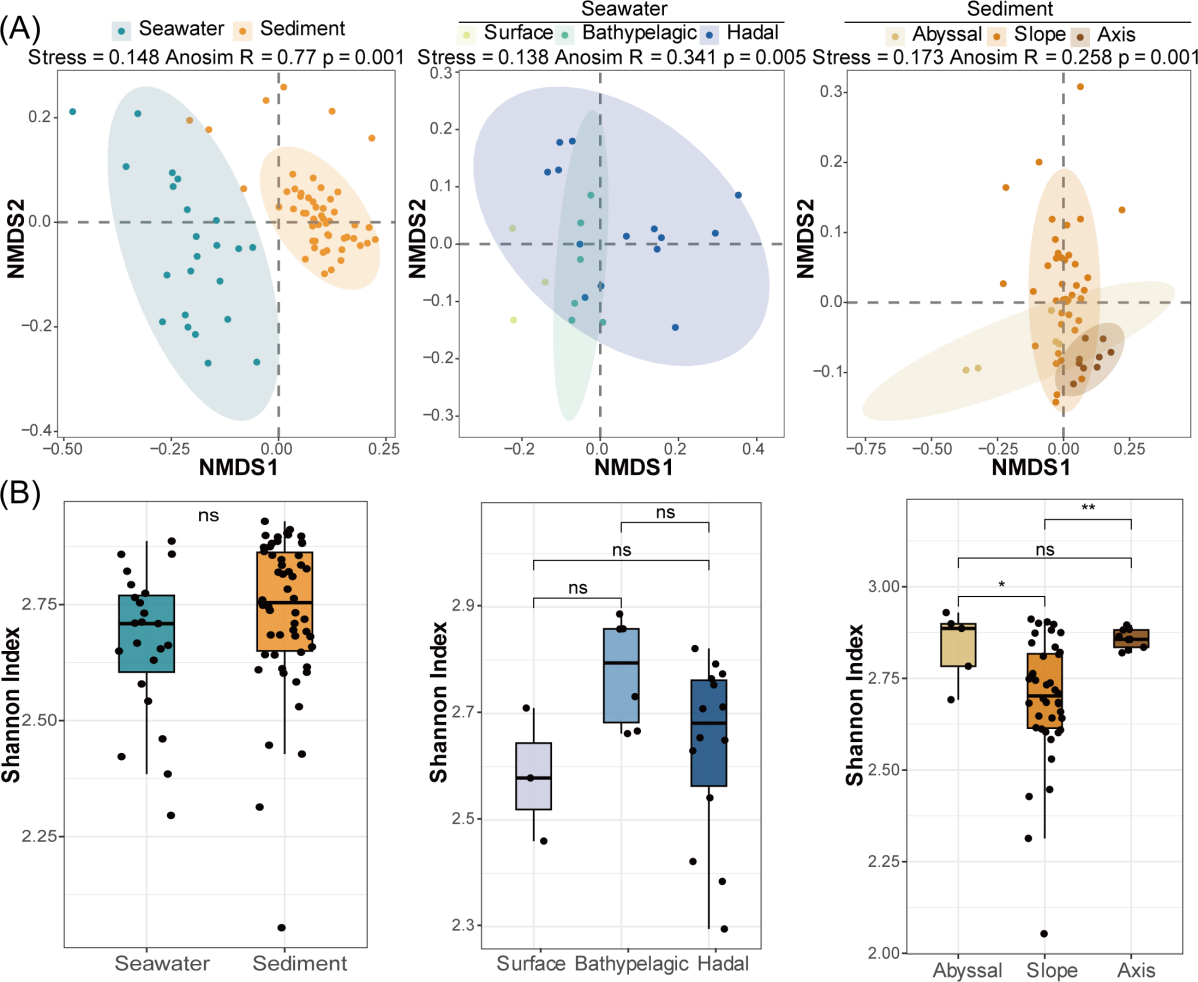
Comparisons of the diversity of D-AA functional genes between different habitats. (**A**) Non-metric multidimensional scaling (NMDS) analysis based on Bray‒Curtis dissimilarities of the composition of D-AA functional genes; the shaded ellipses represent the 95% confidence intervals. (**B**) Alpha diversity of D-AA functional genes; statistics were based on the Wilcoxon test. *P < 0.05, **P < 0.01. ns, no significance.

To reflect the diversity and differentiation among samples, the relative abundance of D-AA functional genes at the KO level (Table S5) was used to calculate the alpha and beta diversity metrics. The samples were grouped according to their water depth and sample type. The seawater samples were divided into three groups: surface (0 m), bathypelagic (2,000–4,000 m), and hadal (>6,000 m). The sediment samples were divided into three groups: abyssal (5,333–6,000 m), slope (6,000–10,000 m), and axis (>10,000 m). The overall D-AA functional gene pool was significantly distinct (analysis of similarities [ANOSIM], *R* = 0.77, *P* = 0.001) between the seawater and sediment in the Mariana Trench (Fig. 2A), whereas no difference was detected in the alpha diversity (Shannon index) of D-AA functional genes (Fig. 2B). Significant separation did not occur among samples grouped by water depth, but bathypelagic and hadal seawaters were more closely related, and sediment samples from the trench axis and slope regions showed greater similarity. The Shannon index did not significantly differ among the different seawater groups, but a significantly lower alpha diversity of D-AA functional genes was detected in the slope sediment than in the other sediment regions (Fig. 2B). These results generally indicate that in terms of D-AA metabolism, the gene composition differed between different habitats, whereas the alpha diversity was relatively consistent.

### Core D-AA metabolic processes in different Mariana Trench habitats

**Fig 3:**
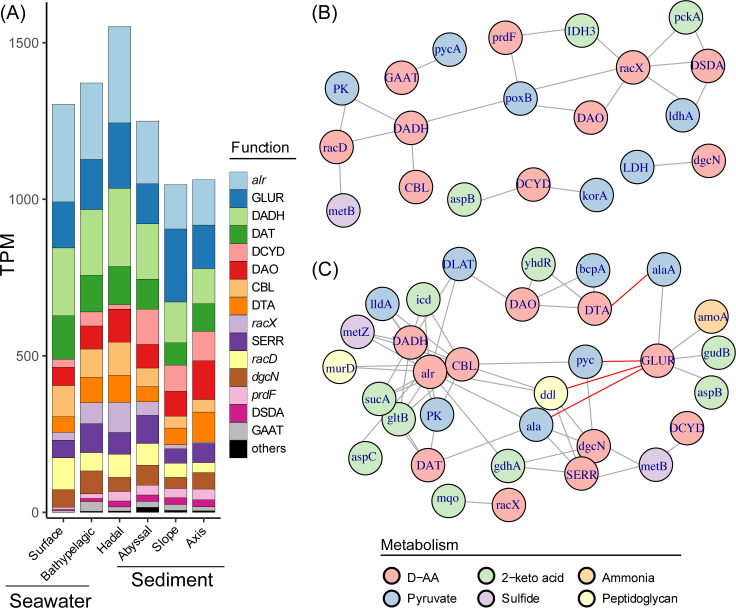
Abundant D-AA functional clusters present in the Mariana Trench metagenome and their potential downstream metabolic processes. (**A**) Mean relative abundance of D-AA functional clusters in specific habitats; clusters with abundances of less than 2% across all the habitats were combined with “others.” (**B**) Co-occurrence network of D-AA functional clusters and potential downstream genes among seawater samples. (**C**) Co-occurrence network of D-AA functional clusters and potential downstream genes among sediment samples. For the co-occurrence network, edges represent Spearman correlations with a |*ρ*| >0.7 (false discovery rate-corrected *P* < 0.001). Gray and red edges represent positive and negative correlations, respectively, and edges between downstream genes are omitted.

The abundance per functional cluster was calculated for the grouped samples to reflect core D-AA metabolic processes in different habitats (Fig. 3A and Table S5). Core genes were defined as those with a mean relative abundance that exceeded 0.01% (mean transcripts per million reads [TPM] > 100) (37) in specific habitats. The D-AA functional clusters are involved in either anabolism or catabolism. Among them, although racemases and D-AA transaminases (DATs) exhibit dual functional associations with both D-AA production and degradation, we primarily discuss them in the context of D-AA anabolism. For D-AA anabolism, alanine racemase (*alr*) and glutamate racemase (GLUR), which are responsible for providing essential D-AA materials for peptidoglycan synthesis, were the most abundant core clusters in all the habitats, with mean TPM ranges of 142–312 and 128–233, respectively. DAT is also abundant and plays an essential role in seawaters (mean TPM >117 in seawater habitats). The core anabolic functional clusters in specific habitats included a broad-spectrum amino acid racemase (CBL) in hadal seawaters (mean TPM = 106) and Asp racemase (*racD*) in surface seawater (mean TPM = 102). Ser racemase (SERR) and broad-spectrum amino acid racemase (*racX*) were also relatively abundant, especially in seawater. For D-AA catabolism, D-AA dehydrogenase (DADH) was most abundant and played a core role in all habitats (with a mean TPM ranging from 112 to 250), followed by D-AA oxidase (DAO), D-Cys desulfhydrase (DCYD), and D-Thr aldolase (DTA). DAO was more crucial in deeper environments, including hadal seawater and axis sediment (mean TPM = 105 and 124, respectively), and DCYD formed a core cluster in abyssal sediment (mean TPM = 113).

### Habitat-specific D-AA metabolic interactions in the Mariana Trench

We further investigated the connection between the D-AA functional clusters and potential downstream genes (Table S3) via co-occurrence network analysis based on Spearman rank correlations of gene relative abundance, to reveal the complete metabolic processes of D-AAs (Fig. 3B and C). Genes involved in the metabolism of potential degradation products of D-AAs (pyruvate, other 2-keto acids, ammonia, and H_2_S) as well as peptidoglycan synthesis are considered potential downstream genes. The results indicated that 10 and 11 of the top 16 clusters presented statistically robust co-occurrence with downstream genes or other D-AA functional clusters in seawater and sediment, respectively.

The co-occurrence of certain metabolic processes generally exhibited similar patterns in both sediment and seawater. The majority of D-AA functional clusters (14 out of 16) were connected positively to pyruvate and other 2-keto acid-related genes (ρ > 0.70, *P* < 2.05e−04), which contribute to further mineralization via central carbon metabolism or the synthesis of other amino acids via transamination. The assimilatory genes for H_2_S (the cystathionine gamma-synthase gene *metB* and O-succinylhomoserine sulfhydrylase gene *metZ*) occurred in the network (ρ > 0.70, *P* < 7.88e−06), indicating that D-AA-derived H_2_S can be reassimilated. Connections among different D-AA functional clusters were also observed, especially between racemases and oxidases/dehydrogenases (ρ > 0.73, *P* < 2.05e−04), which can be understood in two ways. First, the production of D-AAs by racemases always induces D-AA degradation in the environment. Second, as reported in a previous study, the interaction of racemases and D-AA dehydrogenases serves as an additional pathway for L-AA catabolism (38).

In contrast, specific correlations occurred exclusively in the sediment. A consortium closely related to GLUR (ρ > 0.78, *P* < 2.71e−11), including the ammonia monooxygenase *amoA*, the L-Glu dehydrogenase *gudB*, and two amino acid transaminases, *alaA* and *aspB*, was observed in the sediment (Fig. 2C), suggesting that 2-ketoglutarate and ammonia produced from D-Glu through racemization and dehydrogenation can generate other amino acids and hydroxylamine through transamination and ammonia oxidation, respectively. In sediment, we also observed negative correlations between GLUR and the peptidoglycan-related gene, *ddl* (ρ = −0.76, *P* = 1.84e−10), suggesting that the role of GLUR extends beyond peptidoglycan synthesis. This relationship was not apparent in the seawater samples, highlighting the potential environmental specificity of the function of GLUR.

### Widely distributed D-AA functional genes in Mariana Trench metagenome-assembled genomes

**Fig 4:**
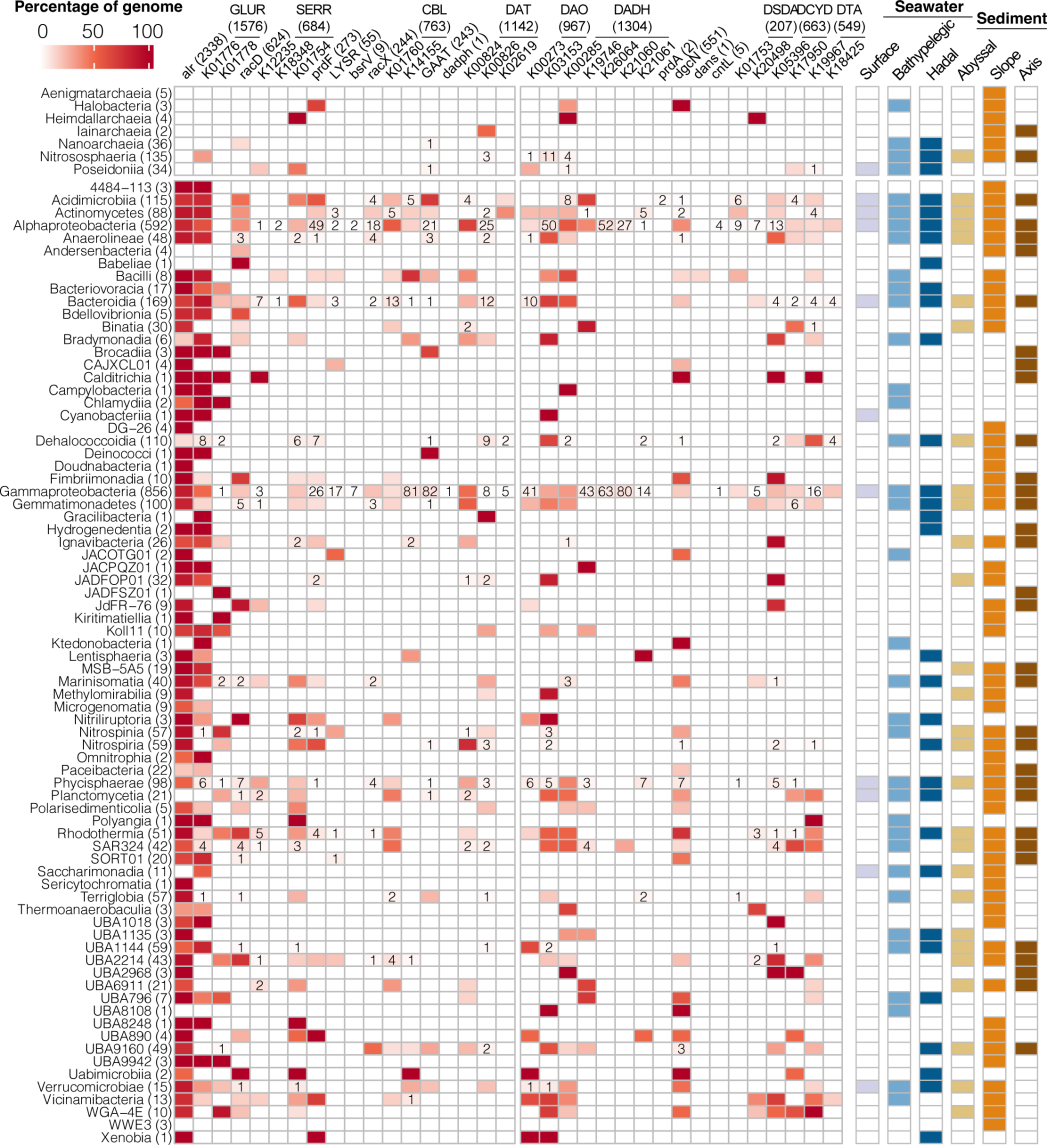
Distribution of D-AA functional genes in MAGs. The heatmap shows the percentage of MAGs containing different D-AA functional genes across the class level. The number of genomes per class and the number of genomes containing each D-AA functional gene are shown in parentheses, and the number of each D-AA functional gene present in <10% of the genomes is shown in squares.

To investigate the microbial taxa that perform D-AA metabolic processes in the Mariana Trench microbiome, we analyzed the distribution of D-AA functional genes at the metagenome-assembled genome (MAG) level (Fig. 4). In total, 3,259 MAGs of medium or higher quality (completeness >50% and contamination <10%) were retained from 78 samples. The obtained MAGs were taxonomically classified and named based on the Genome Taxonomy Database (GTDB, release version 220) and were assigned to 83 known classes, including 7 classes of archaea, such as Nitrososphaeria, Nanoarchaeia, and Poseidoniia, and 76 classes of bacteria, dominated by Alphaproteobacteria and Gammaproteobacteria.

When D-AA functional genes were annotated to each MAG, 93.6% of the MAGs contained at least one D-AA functional gene, covering 81 different class-level clades (Fig. 4 and Table S6), with an average of 4.58 D-AA functional genes per MAG and an average gene density of 1.81 Mbp^−1^. Excluding *alr* and GLUR, which are relatively conserved for peptidoglycan biosynthesis, 84.7% of the MAGs contained at least one D-AA functional gene, accounting for 67 class-level clades. Bacteria are the primary sources of D-AA functional genes, the number and density of which are 8.0 and 4.1 times greater than those in archaea, respectively. However, we still identified 12 D-AA functional clusters in the archaeal MAGs of this study. Genes mapped in MAGs (approximately 41% of the total abundance of D-AA functional genes) were used to reflect and compare the composition of microbial taxa and functional clusters related to D-AA metabolism in different samples. The relatively stable composition of D-AA functional clusters (ANOSIM, *R* = 0.162, *P* = 0.015) was similar to that at the contig level, whereas the taxonomic composition was more variable (ANOSIM, *R* = 0.351, *P* = 0.001) among different habitats (Fig. S4 to S6). This evidence collectively demonstrates that D-AA metabolism is a ubiquitously distributed fundamental ecological function performed by highly diverse microbial groups across different environments.

In terms of D-AA anabolism, the two core racemases, *alr* and GLUR, exhibited the widest distributions in class-level clades of our MAGs and covered most bacterial clades. The absence of *alr* and GLUR is lethal for bacteria when no additional D-Ala or D-Glu is present (18, 39) because of their crucial role in providing D-Ala and D-Glu for peptidoglycan synthesis. Therefore, bacteria lacking both *alr* and GLUR, or one of them and DAT, are more likely to recycle environmental D-AAs than to produce them. By further examining the presence of DAT as well as downstream genes for peptidoglycan synthesis, namely, *ddl* and *murD* (which mediate the synthesis of D-Ala dimers and UDP-N-acetylmuramoyl-L-ala-D-glu, respectively), we found that some minor classes, such as Planctomycetia, Acidobacteriae, Nitrospinia, SAR324, and Binatia*,* may be important for D-AA recycling.

We also detected other racemases in 56.7% of the MAGs across 50 classes of bacteria and archaea. These racemases included four substrate-specific racemases and four broad-spectrum racemases, with the more common *racD*, SERR, and CBL found in 19.1%, 21%, and 23% of the MAGs, respectively. These racemases are widely distributed among various dominant or minor class-level clades, with Pseudomonadota (Alphaproteobacteria and Gammaproteobacteria), Actinomycetota (Actinomycetes and Acidimicrobiia), and Bacteroidetes (Bacteroidia and Rhodothermia) being significant sources of multiple racemases. Specific racemases were distributed in UBA2214 (*racD*), Phycisphaerae (SERR), Nitrospiria (SERR and *prdF*), Nitrospinia (LYSR), UBA9160 (*racX*), and Terriglobia (GAAT). Similarly, compared with the Tara Oceans microbiome and marine bacterial genomes from the IMG/JGI database, where Gammaproteobacteria predominantly contribute to DAT (28), our MAG-based analysis revealed a broader distribution of DAT in the Mariana Trench microbiome, with Alphaproteobacteria, Nitrospiria, and Gemmatimonadetes also widely encoding DAT.

For D-AA catabolism, two oxidases of D-AAs, DADH and DAO, were the most widely distributed functional clusters and were present in 58.8% of the MAGs. Moreover, multiple copies of D-AA-related oxidases were present in 29.1% of the MAGs, which may be important for microbes to extend their repertoire of utilizable D-AAs. The two oxidases were both common in the dominant taxa. DADH was more predominant in Gammaproteobacteria and Alphaproteobacteria*,* and a greater proportion of DAO was found in Bacteroidia, Chloroflexota (Anaerolineae and Dehalococcoidia), and Gemmatimonadetes. The functional clusters with the second widest occurrence were DCYD, *dgcN*, and DTA (Fig. 4; Fig. S7), which were distributed in 33, 32, and 26 class-level clades, respectively. *dgcN* is the key gene of a newly identified irreversible racemization pathway for D-type acidic amino acids and their amides (Fig. 1). Compared with the *dgcN* identified in the Tara Oceans and polar metagenomes, which mostly belong to Alphaproteobacteria (27), we found that Bacteroidetes (Bacteroidia and Rhodothermia), Marinisomatia, and Gammaproteobacteria (Woeseiales) are also significant carriers of *dgcN* in the Mariana Trench. DCYD was widespread in Gammaproteobacteria, notably within Woeseiales and Acidiferrobacterales, as well as in Rhodospirillales of Alphaproteobacteria, UBA2214, and SAR324. DTA was generally distributed in Dehalococcoidia, as well as in specific classes of Gammaproteobacteria and Alphaproteobacteria (Pseudomonadales, Rhodobacterales, and Nevskiales).

### Unexpected prevalence and potential metabolic role of glutamate racemase in ammonia-oxidizing archaea

**Fig 5:**
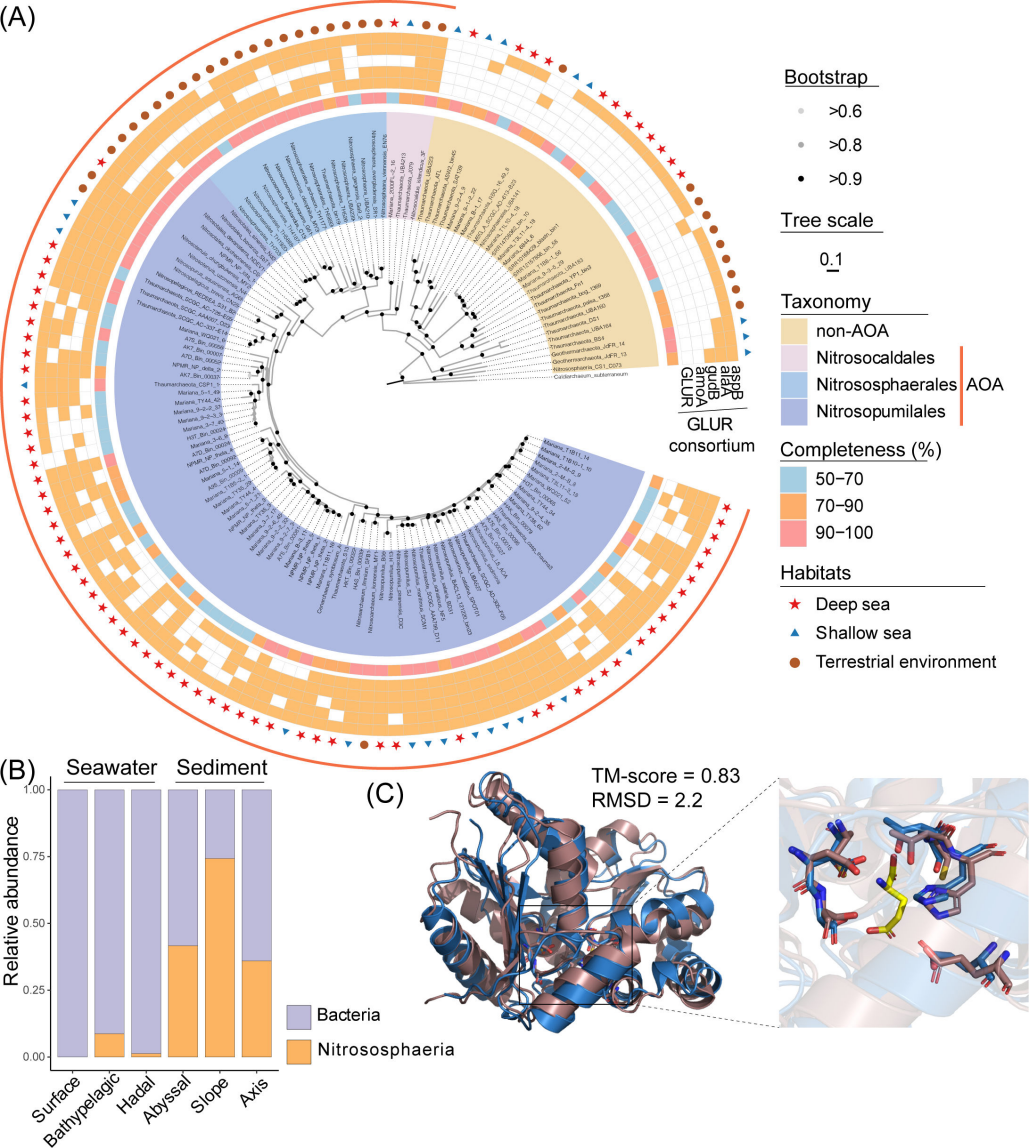
The GLUR of AOA is an important component of the GLUR in the deep sea. (**A**) Distribution of the GLUR consortium in Nitrososphaeria. (**B**) Taxon assigned for GLUR in the Mariana Trench metagenome. (**C**) Structure alignment between GLUR_Nm (blue) and 2GZM (purple); the zoomed-out image on the right shows D-Glu (yellow) and the active site residues. RMSD, root mean square deviation.

Interestingly, although *alr* and GLUR are thought to be bacteria-specific genes since peptidoglycan is absent in archaea, the typical bacterial GLUR (K01776) was detected in 31.0% of the Nitrososphaeria MAGs. Moreover, it made a significant contribution to the GLUR in deep-sea samples, especially in sediment, where the GLUR of Nitrososphaeria represented 32.2%–72.4% of the total GLUR (Fig. 5B). Comparative analysis of 134 duplicated medium- or high-quality genomes of Nitrososphaeria, including 97 from public databases, revealed that GLUR is prevalent among different clades of ammonia-oxidizing archaea (AOA), whereas it is virtually absent in non-AOA Nitrososphaeria MAGs (Fig. 5A). The predicted structure of GLUR_Nm (GLUR of the type AOA strain *Nitrosopumilus maritimus* SCM1) indicated that GLUR_Nm highly matched the structure of GLUR from *Bacillus anthracis* (TM score = 0.83, root mean square deviation = 2.2) and had the necessary catalytic active sites and conserved residues involved in the deprotonation of glutamate enantiomers (40) (Fig. 5C). Phylogenetic analysis revealed that the GLUR of AOA was derived from bacteria (Fig. S8). The wide distribution of GLUR and genes from the GLUR-related consortium (*amoA*, *gludB*, *alaA*, and *aspB*) in AOA MAGs provided a good explanation for their co-occurrence and further indicated the key role of AOA GLUR in expanding the capacity to utilize D-Glu as a carbon or ammonia source.

### Spatial distribution of microbial D-AA metabolism in the Mariana Trench

To further elucidate the metabolic processes driving D-AA metabolism differentiation across habitats, we conducted two comparisons: (i) near-bottom seawater and sediment (depths greater than 10,000 m) and (ii) seawater or surface sediment across different depths (only surface sediment samples are considered for comparison to minimize the influence of other factors like redox gradients). These analyses were based mainly on the relative abundance (TPM) of the top 16 clusters. The absolute abundance and proportion of bacterial cells harboring these clusters were also assessed to provide more comprehensive comparisons.

### Comparison of the near-bottom seawater and sediment

**Fig 6:**
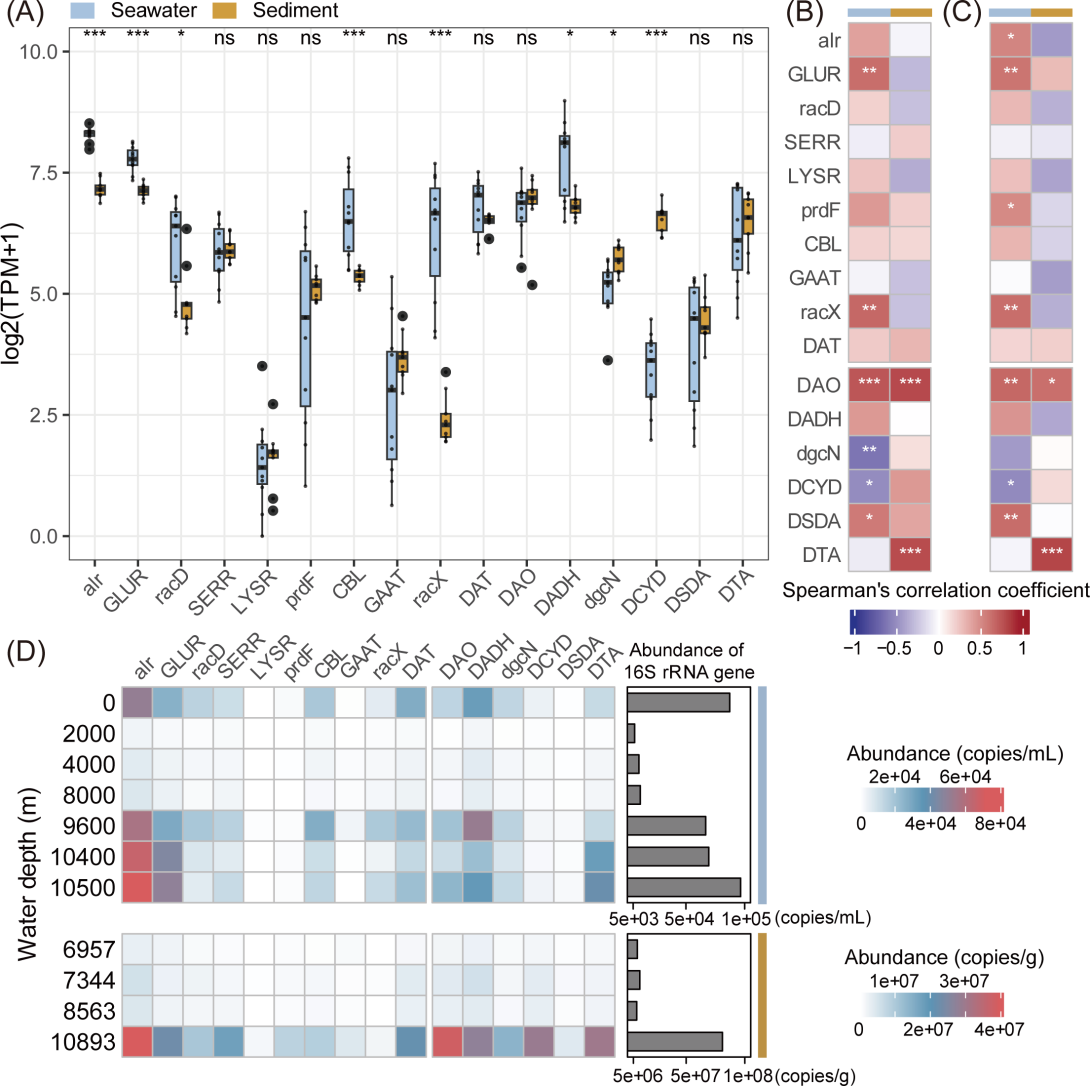
Statistical analysis reflecting the spatial variation in D-AA functional genes in the Mariana Trench. (**A**) Comparisons of the relative abundances of different D-AA functional clusters between seawater and sediment near the bottom. Statistics were based on the Wilcoxon test. (**B**) Spearman correlation analysis between the relative abundance of D-AA functional clusters and depth. (**C**) Spearman correlation analysis between the proportion of bacterial cells harboring D-AA functional clusters and depth. (**D**) Copy numbers of 16S rRNA and D-AA functional clusters at different depths. For all the statistical analyses, *P* values were corrected via the false discovery rate method. *P < 0.05, **P < 0.01, ***P < 0.001.

Comparative analysis revealed distinct environmental partitioning of D-AA metabolic potential between the near-bottom seawater and sediment microbial communities. Half of the D-AA functional clusters showed significant differences, particularly those involved in biosynthesis (Fig. 6A). Most of the anabolic clusters were more abundant in seawater, including those with core roles (*alr*, GLUR, CBL, and *racX*). With respect to catabolism, the dominant catabolic cluster DADH was slightly enriched in seawater. The opposite tendency was observed for DCYD and *dgcN*. The proportion of bacterial cells harboring these genes exhibited similar trends (Fig. S9). These results indicated an overall greater potential for D-AA metabolism in the seawater microbial community, especially for D-AA production.

MAG-mapped genes were analyzed to identify the microbial taxa contributing to D-AA metabolism in sediment or seawater (Fig. S10). The enrichment of most functional clusters, including *alr*, GLUR, CBL, *racX*, and DADH, in seawater was predominantly attributed to heterotrophic bacteria within Gammaproteobacteria and Alphaproteobacteria, particularly Pseudomonadales and Enterobacterales. Moreover, the enrichment of *racD* in seawater was driven mainly by Alphaproteobacteria and Actinomycetes. In contrast, the enrichment of DCYD in the sediment was associated primarily with Woeseiales, Gemmatimonadetes, Dehalococcoidia, and UBA2214. These results suggest a distinct preference for D-AA metabolism among the microbial taxa in seawater and sediment.

### Comparison of seawater or surface sediment across different depths

The Spearman correlation coefficient between water depth and the relative abundance of D-AA functional genes was calculated to assess their distribution across depth strata (Fig. 6B). In the seawater samples, most functional clusters exhibited relatively high abundances in deeper seawater, with a significant increasing trend observed for GLUR, *racX*, DAO, and DSDA. In contrast, the abundances of *dgcN* and DCYD significantly decreased with increasing water depth. Notably, the proportions of bacterial cells harboring the two core racemases, *alr* and GLUR, followed a similar pattern (Fig. 6C), increasing from 0.56 to 0.77 and from 0.27 to 0.52, respectively, from the surface to the hadal seawater. In the surface sediment, most of the catabolic functional clusters tended to increase with depth, with DAO and DTA showing significant correlations.

To further investigate the microbial taxa contributing to the increased D-AA metabolic potential in hadal microbial communities at the MAG level, we examined the taxonomic distribution of D-AA functional genes across samples from different depths (Fig. S11). In hadal seawater, Actinomycetes, Gammaproteobacteria (primarily Pseudomonadales and Enterobacterales), and Bacteroidia were the dominant contributors to the enrichment of D-AA functional genes. Specifically, Pseudomonadales, Actinomycetes, and Bacteroidia were associated with the enrichment of GLUR and DAO, whereas Actinomycetes also contributed to the enrichment of DSDA. The enrichment of *racX* was linked primarily to Pseudomonadales and Enterobacterales. In the surface sediment, Gemmatimonadetes and Dehalococcoidia were the major contributors to the increase in the abundance of DAO in axis sediments, whereas Dehalococcoidia was also associated with the enrichment of DTA in these sediments. At the contig level, we assessed the proportions of *alr*- and GLUR-containing cells among the dominant bacterial classes in seawater (Fig. S12). The results revealed a significant increase in the proportion of GLUR-containing Bacteroidia, Gammaproteobacteria, and Alphaproteobacteria with increasing water depth. Similarly, the proportion of Alphaproteobacteria encoding *alr* also significantly increased with depth.

### Spatial distribution of microbial D-AA metabolism on the basis of absolute quantification

To provide more direct evidence of the spatial distribution of D-AA metabolism, the absolute abundance of genes was estimated by multiplying the microbial biomass by the proportion of cells harboring the target genes (Fig. 6D; Table S7). Microbial biomass was determined based on 16S rRNA gene quantification from either this study or previously published data (41, 42), while the proportion of gene-containing cells was calculated by normalizing gene abundance to the single-copy gene *recA* (see Materials and Methods for details).

In the water column, surface samples (8.73 × 10^4^ 16S rRNA gene copies/mL) and those from depths exceeding 9,600 m (6.68 × 10^4^ to 9.66 × 10^4^ 16S rRNA gene copies/mL) exhibited substantially greater microbial biomass than mid-depth samples between 2,000 and 8,000 m (6.26 × 10^3^ to 1.11 × 10^4^ 16S rRNA gene copies/mL). Consequently, these high-biomass regions also demonstrated elevated absolute abundances of D-AA functional genes. Most notably, we consistently observed peak abundances of DSDA and two core racemases, *alr* and GLUR, in the three deepest seawater samples. The absolute abundances of these genes reached 8.64 × 10^2^ to 1.44 × 10^3^, 5.69 × 10^4^ to 7.14 × 10^4^, and 2.83 × 10^4^ to 4.92 × 10^4^ copies/mL, respectively, in the deepest samples, representing levels that were on average 3.63, 1.27, and 1.58 times higher than those in surface waters. Similarly, in the surface sediment, the axis sample (8.10 × 10^7^ 16S rRNA gene copies/g) presented significantly greater microbial biomass than the three slope samples (7.83 × 10^6^ to 1.05 × 10^7^ 16S rRNA gene copies/g), with a corresponding enrichment of D-AA functional genes in the deepest sediment sample. These findings collectively suggest that both near-bottom seawater and trench sediments play critical roles in D-AA metabolism, potentially contributing to the maintenance of elevated microbial biomass in these extreme habitats .

## DISCUSSION

In this study, we observed widespread and diverse D-AA functional genes in the microbiomes of seawater and sediment from different depths in the Mariana Trench. Overall, similar D-AA metabolic processes were performed by highly variable microorganisms in different habitats, demonstrating that both D-AA production and degradation are essential ecosystem functions. The widespread D-AA production processes meet the fundamental need of bacteria for peptidoglycan modification, a phenomenon observed across all bacteria examined in previous studies (16, 24, 29, 36). In contrast, the great potential of microbes in D-AA degradation challenges the common perception that D-AAs are recalcitrant. The assessment of D-AA recalcitrance is typically based on their longer persistence in the environment than that of L-AAs (36, 43), and microorganisms generally exhibit a lower capacity to utilize D-AAs than L-AAs do (28, 44). However, catabolic genes for D-AAs are more abundant than those for other recalcitrant organic compounds (such as aromatic compounds, alkanes, and complex sugars [33]) in the hadal zone. This may be due to the preference of microorganisms for nitrogen-containing organic matter (11), as well as the relatively simple structure of D-AAs, which does not require overcoming significant energy barriers. Moreover, considering the toxicity of D-AAs in interfering with protein and peptidoglycan synthesis (45, 46), the prevalence of D-AA catabolic genes may also be a response to ubiquitous toxic D-AAs, such as DCYD for D-Cys. Therefore, although D-AAs are more resistant to degradation than labile organic matter, such as L-AAs, the widespread presence of D-AA catabolic genes suggests that D-AAs could serve as important nutrients, particularly in deep-sea environments where RDOM accounts for a much greater proportion of the organic carbon pool than the upper ocean.

Furthermore, compared with culture-based studies, genomic data mining has significantly expanded our understanding of microbial groups with the potential capability for D-AA utilization (13, 14, 44, 47). The widespread distribution of D-AA catabolic genes among dominant hadal microbial groups may considerably influence the remineralization of D-AAs as both carbon and nitrogen sources. A significant case is AOA. AOA are ubiquitous from shallow seas to deep seas and have been identified as one of the predominant groups in hadal seawaters and sediments; AOA can fix CO_2_ by generating reducing forces during the ammonia oxidation process and therefore play a significant ecological role in both carbon and nitrogen cycling (48, 49). Our results revealed that the conserved bacterial GLUR (K01776) is ubiquitous in various clades of AOA but not in non-AOA Nitrososphaeria and that AOA-derived GLUR significantly contributes to the deep-sea GLUR pool. The acquisition of GLUR more likely allows AOA to utilize D-Glu as a nitrogen and carbon source rather than produce it on the basis of the following considerations. First, no peptidoglycan-like component has been found in archaea, and bacterial D-AAs are estimated to be almost exclusive sources of marine D-AAs (16). The acquisition of GLUR, similar to genes involved in amino acid metabolism and ammonia oxidation (50, 51), occurred during the evolutionary transition from non-AOA Nitrososphaeria to AOA, which indicates that GLUR may also facilitate ammonia oxidation. Moreover, horizontally transferred *alr* from bacteria enabled *Methanococcus maripaludis* to utilize D-Ala as a nitrogen source (52), further suggesting that GLUR in AOA may be involved in D-Glu catabolism. A previous study also indicated that the utilization of amino acids such as glycine and serine is an important strategy of deep-sea AOA (53). Together, the abundance of functional genes and the diversity of participants indicate that there has been an underestimation of D-AA metabolic processes, especially the degradation of D-AAs.

Although similar D-AA metabolic processes occurred in different habitats, specific D-AA functional genes present significant enrichment in the deepest regions of the Mariana Trench, both in terms of relative and absolute abundance. This enrichment is driven by dominant microbial taxa in both hadal seawater and sediments, further suggesting a strong link between these genes and adaptation to the hadal environment. This adaptive advantage likely stems from the diverse physiological roles of D-AAs (54), which include their necessity for peptidoglycan modification, their ability to respond to environmental stress, and their ability to serve as carbon and nitrogen sources. We discuss the distribution patterns of D-AA functional genes driven by these roles in the subsequent discussion.

On the basis of the evidence that D-AAs are less available in deeper seawater (16), the greater abundance of core racemases (*alr* and GLUR) in hadal seawater than in upper seawater may be due to the fundamental role of D-Ala and D-Glu in peptidoglycan synthesis. We found that the enhanced potential for D-AA production as an adaptive strategy is present across three dominant microbial taxa (Alphaproteobacteria, Gammaproteobacteria, and Bacteroidia) in hadal seawater, further supporting its role as a common microbial adaptation to environments with limited D-AA availability.

The important role of D-AAs in adaptation to environmental stress may also promote their production in hadal environments. The enrichment of the broad-spectrum racemase *racX* in hadal seawater is driven primarily by Pseudomonadales and Enterobacterales. Although the physiological functions of *racX* in these taxa remain uncharacterized, accumulating evidence suggests that non-canonical D-AAs biosynthesized through such enzymes are associated with multifaceted environmental adaptation mechanisms for these taxa. The produced D-AAs can be utilized for the synthesis of NRPs (22, 23), providing an advantage in nutrient competition, which is more abundant in deep-ocean communities (55). Moreover, the great potential for the production of NCDAAs may also benefit the regulation of peptidoglycan strength (24) in response to environmental stress, as well as function as chemotaxis signaling molecules (25) to allow exploration of favorable ecological niches.

The contribution of D-AAs to total organic matter increases with water depth in seawater, as reflected by the higher D/L-AA ratio (16, 43), highlighting the increased contribution of D-AAs to the deep-sea organic carbon pool. Building upon these observations, our metagenomic results revealed an enrichment of racemases, further reinforcing the importance of D-AAs in hadal seawater. Two additional factors could further contribute to the increased contribution of D-AAs to the hadal organic carbon pool: (i) viral shunt-mediated release through elevated virus:prokaryote ratios (56, 57) and increased expression levels of cell wall‒lytic enzymes (M23 peptidases/GH23 lyases) (58) and (2) geochemical contributions via abiotic racemization in aged particulates and subduction zone-specific synthesis (59). The increased catabolic potential of D-AAs may be promoted by their increased contribution to the hadal seawater, dominated by Actinomycetes, Gammaproteobacteria, and Bacteroidia. These taxa exhibit metabolic capabilities for degrading recalcitrant organic matter (32, 42). Notably, Actinomycetes significantly contribute to the production of both M23 peptidase and GH23 lyase (58), suggesting their comprehensive cell wall degradation capacity in the hadal environment.

Moreover, surface sediments from greater water depths exhibited elevated abundances of D-AA catabolic genes, primarily attributed to the class Dehalococcoidia within Chloroflexi. This pattern likely reflects the combined effects of increasing recalcitrance of organic carbon supplied from the overlying water column with water depth (60) and the dynamic sedimentation regime characteristic of trench environments. Frequent lateral transport processes deliver pre-aged organic matter from trench slopes to the axis while simultaneously burying fresher material into deeper sediment layers, thereby enriching surface sediments with recalcitrant compounds (5, 7). The dominance of Chloroflexi in axis surface sediments aligns with the phylum’s well-documented capacity to degrade diverse recalcitrant organic compounds, positioning it as a key player under the fluctuating organic matter inputs typical of hadal environments (8). Our findings reveal that D-AA catabolism represents an additional dimension of Chloroflexi metabolic versatility, expanding its known repertoire of strategies for utilizing recalcitrant organic matter in these extreme hadal habitats.

The potential utility of D-AA metabolites—such as L-AAs, 2-keto acids, and H_2_S—in deep-sea adaptation may also explain the enhancement of D-AA catabolic pathways in these environments. The deep sea, particularly the hadal trench environment, is well known for its cold and high-pressure conditions. Two universal strategies for microorganisms to cope with low temperature and high pressure are antioxidation and the accumulation of small molecules such as amino acids as compatible solutes to regulate osmotic pressure (61). A previous study indicated that L-AAs but not D-AAs can act as compatible solutes (62). The presence of numerous racemases provides an additional way for deep-sea microbes to accumulate compatible solutes such as L-Glu, L-Pro, and L-Ala. As a core strategy for coping with multiple types of environmental stress (2), the enhanced capacity for antioxidation has been shown to help microorganisms tolerate low temperatures and high pressure (63). H_2_S produced by the decomposition of sulfur-containing D-AAs can help cells combat oxidative stress by reacting with reactive oxygen species (64) or increasing the expression of key antioxidants such as catalase and superoxide dismutase (65, 66). The universal metabolites of D-AAs, namely, 2-keto acids, can serve as direct scavengers of H_2_O_2_ (67).

In contrast, the role of D-AA catabolism in detoxification may decrease as its concentration decreases. DCYD is considered a specific detoxification gene for D-Cys and has been proven to be more toxic to cells than other D-AAs (68), and knockout of DCYD increases the sensitivity of *Escherichia coli* to D-Cys (69). A trend toward depletion of DCYD with increasing water depth was observed, similar to the D-AA concentration. A greater abundance of DCYD was also observed in sediments with higher D-AA concentrations than in seawater. In addition, the variation trend of *dgcN* was similar to that of DCYD. *dgcN* is a key gene in a complex D-Glu racemization pathway that involves considerable energetic cost (27). The enrichment of the direct D-Glu racemization pathway (GLUR) in hadal environments may serve as a substitute for this relatively uneconomical metabolic strategy.

**Fig 7:**
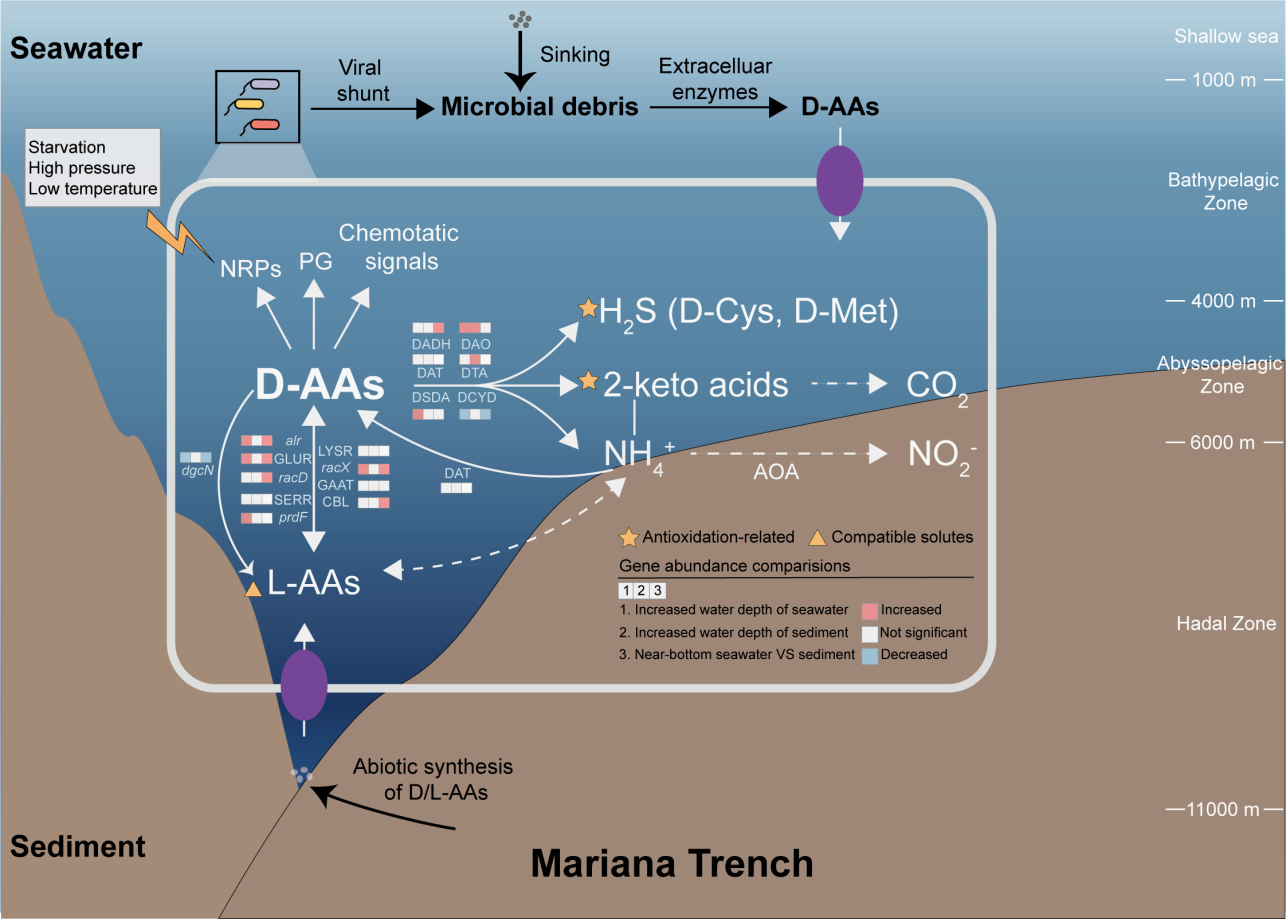
Prokaryotes are both the major contributors to and utilizers of D-AAs. In the deep-sea environment, D-AA levels decrease; however, the contribution of D-AAs to total organic matter increases, as reflected by a higher D/L-AA ratio. The decrease in accessible D-AAs from the environment promotes the potential for D-AA synthesis in the deep sea to meet the essential peptidoglycan synthesis requirements and respond to environmental stress. The increase in endogenous D-AA production, along with other microbial activities, such as viral lysis and the extracellular enzymatic degradation of peptidoglycan, as well as the increase in D-AAs abundantly sourced from dark environments, collectively contribute to the enhanced role of D-AAs in the deep-sea organic carbon pool, making them crucial carbon and nitrogen sources for microorganisms. Similarly, compared with those in sediment, the lower concentrations of D-AAs and the higher D/L-AA ratios in seawater suggest a potentially faster D-AA turnover rate in the water column. PG, peptidoglycan.

Overall, to increase the utilization of recalcitrant organic matter, fulfill the essential need for peptidoglycan synthesis, and adapt to the low-temperature and high-pressure conditions of the deep sea, an increase in both D-AA production and degradation was observed in the hadal trench compared with those in the upper ocean (Fig. 7). These microbial processes may ultimately contribute to greater D-AA turnover rates in deeper marine environments. On the other hand, due to the higher concentrations of D-AAs and lower D/L-AA ratios in sediments (29, 70, 71), D-AA metabolism, particularly the anabolism of D-AAs, is less abundant than that in seawater (Fig. 7), indicating a more significant role of seawater microbes in D-AA turnover.

In summary, our findings provide genetic profiles of D-AA metabolism in marine environments, especially in deep-sea ecosystems, and preliminary insights into the production and remineralization processes of D-AAs. These processes are commonly performed by various marine bacteria and archaea. The differentiation of the D-AA functional gene pool occurred in different habitats, which indicates that both production and degradation were intensified in deeper marine environments (based on water depth profiles) and seawater (based on comparisons between seawater and sediment). Our study highlights the important role of deep-sea microbes in D-AA turnover, which needs further attention, such as exploration via multiomics methods, as well as the extension of the D-AA profile to deeper environments, such as hadal trenches. Finally, it is crucial for future studies to consider both the production and degradation rates of D-AAs, as well as other RDOMs, to better understand their fluxes and thus their significance in marine environments.

## MATERIALS AND METHODS

### Data set collection

During the TS-09 cruise aboard the R/V “Tan Suo Yi Hao” in September and October 2018, four sediment samples were collected from the Mariana Trench using a push corer deployed by the Tianya deep-sea lander. The TS-09 cruise was conducted with all necessary permissions for marine scientific research in the relevant waters in accordance with international law. All sediment samples were frozen at −80°C immediately before DNA extraction. Genomic DNA was extracted from 1 to 5 g of sediment via a refined SDS-based method (72). The samples were mixed with 0.1 and 0.5 mm glass bead mixtures and extraction buffer by low-speed vortexing. The mixture was homogenized with an MP FastPrep-24 homogenizer (MP Biomedicals, California, USA). Lysozyme and proteinase-K were added, and the mixture was incubated for 30 min at 37°C. Then, 20% SDS was added, and the mixture was incubated at 65°C for 2 h. The supernatant was collected by centrifugation at 10,000 × *g* for 10 min. The extraction was repeated once with residual pellets. The supernatants from the two rounds of extraction were combined and mixed with equal volumes of phenol:chloroform:isoamyl alcohol (24:25:1 [vol/vol], pH 8.0). The aqueous phase was recovered by centrifugation and then precipitated overnight at 4°C with 0.6 vol of isopropyl alcohol and 0.1 vol of 3 M sodium acetate (pH 5.2). The pellet was collected and washed twice with pre-cooled 70% ethanol and then dissolved in Tris-EDTA buffer. The DNA was further purified with a Boreal Genomics Aurora nucleic acid extraction system (Boreal Genomics, Vancouver, BC, Canada) (73). A total of 50–100 ng of DNA was used for library construction via the MGIEasy DNA Library Prep Kit (MGI, China), and shotgun sequencing was performed via an Illumina HiSeq X Ten instrument with paired-end 150 bp reads at BGI Co., Ltd. (Shenzhen, China). In addition, 74 metagenomic data sets of the Mariana Trench were downloaded from the European Nucleotide Archive database (31–34). The corresponding metadata (geographical coordinates, water depths, sample types, accession numbers, habitat groupings, and references) of the metagenomes generated in this study, as well as those obtained from public databases, are summarized in Table S4.

### Metagenomic data processing and MAG analysis

The raw data obtained from public databases were initially processed via fastp (version 0.23.4) (74) for quality control. Sequences containing low-quality bases (*Q* < 20) and ambiguous bases (*N* > 20) were removed, and trimmed sequences longer than 90 bp were retained. Clean reads were assembled via Megahit (version 1.2.9) (75) with “--presets meta-sensitive,” and contigs shorter than 1,000 bp were discarded. Read mapping was performed with MetaWRAP (version 1.3) (76), which was also used for binning and optimization; ultimately, 3,259 MAGs with completeness of >50% and contamination of <10% were selected for further analysis. Quality assessment and taxonomic classification of the MAGs were performed via CheckM (version 1.1.3) (77) and GTDB-Tk (version 2.4.0) (78), using version 220 of the GTDB, with taxonomy and naming assigned according to the GTDB classification system.

### Gene annotation and abundance calculation

All genes on the contigs were predicted via Prodigal (version 2.6.3) (79), and those shorter than 99 bp (33 amino acids) were discarded. The relative abundance of genes was subsequently calculated for all the predicted genes. Gene coverage was calculated via featureCounts (version 2.0.1) (80), and abundance was estimated and normalized as follows: TPM = 10^6^ × (mapped reads/gene length)/∑ (mapped reads/gene length). All the predicted genes were subjected to both taxonomic annotation and functional annotation without clustering. Taxonomic annotation was performed via DIAMOND (version 2.0.14.152) (81), aligned against the GTDB (version 220) with an identity of >30% and query coverage of >70%. Functional annotation was primarily performed via KofamKOALA (version 1.3.0, database version 2024-03-26) (82), whereas unannotated genes were subsequently aligned with the UniProt database (version 2023.05) via DIAMOND with the aforementioned threshold.

The proportion of bacterial cells harboring D-AA functional genes was estimated by comparing the TPM of the functional gene to the TPM of the single-copy gene *recA*, which is conserved in bacteria. The absolute abundance of D-AA functional genes was estimated as follows: gene copy number = (copy number of the 16S rRNA gene) × (TPM of the functional gene/TPM of *recA*). For seawater samples, the copy numbers of the 16S rRNA gene were obtained from published studies (41, 42). For the sediment samples, the bacterial 16S rRNA gene was quantified using quantitative PCR (qPCR) with the primers 341F/519R (5-CCTACGGGWGGCWGCA-3/5-TTACCGCGGCKGCTG-3). Genomic DNA for qPCR was extracted and purified using the DNeasy PowerSoil Pro Kit (Qiagen, USA) following the manufacturer’s protocol. The plasmids with the bacterial 16S rRNA gene were used for standard curve construction. qPCR was performed on a StepOnePlus Real-Time PCR System (Applied Biosystems, USA) with PowerUp SYBR Green Master Mix (Applied Biosystems).

### Reference database curation and annotation of D-AA functional genes

A data set containing KOs and identified protein sequences involved in D-AA metabolism was developed. In total, 25 relevant KOs (KO-25, Table S1) were retrieved by searching keywords related to D-AAs (including D-amino acids, amino acid racemases, specific D-AAs, and specific AA racemases) from the KEGG database (https://www.genome.jp/kegg/kegg2.html). Literature searches were performed using the aforementioned keywords across the Google Scholar and PubMed databases to retrieve accession information for 151 proteins (Seq-151) with solid experimental evidence of their D-AA-relevant functions. Detailed information for Seq-151, including references, accession numbers, and substrate/product ranges, is provided in Table S2. The searches were restricted to peer-reviewed publications written in English. The sequences of Seq-151 were retrieved from the NCBI GenBank and UniProt databases.

The KO assignment for Group II of Seq-151 included 17 additional D-AA-related KOs. These KOs are solely used for classifying the genes annotated by Seq-151, and not all sequences assigned to these KOs are accepted as D-AA functional genes. We further clustered all D-AA-related KOs with similar functions to facilitate analysis from the perspective of function. An exception is broad-spectrum racemases. Although they have similar functions, KOs involving broad-spectrum racemases share low homology, as reflected by different features, such as the dependence on coenzymes or whether they are multifunctional enzymes with expanded amino acid racemization activity. The broad-spectrum racemases were divided into four cluster-level units on the basis of their homology. For individually clustered KOs, we used their names from the KEGG database for description. These KOs, together with their functional clusters, assist in describing D-AA metabolism at the functional level. The clusters were all named in uppercase to distinguish them from genes named in lowercase or partial lowercase. The details of the KO list, cluster list, and mapping correlation are described in Table S1.

The annotation and filtering process for D-AA functional genes was as follows:

- Annotation with Seq-151 was performed via DIAMOND with an identity of >30% and query coverage of >70%, and sequences aligned with non-functional proteins were excluded. Strict filtering was subsequently applied, retaining only the subjects that matched the KO assigned to the query or subjects without an assigned KO.
- KO-25 was selected from the results of functional gene annotation via the KofamKOALA or UniProt database and integrated with the results from the last step. In cases of duplicate annotations, priority was given to annotations obtained through alignment with Seq-151.

### Phylogenetic analysis

For the phylogenetic analysis of K01776, the complete protein sequences were retrieved from the metagenomes of our study, along with 13 functionally verified sequences as references and *racD* as the outgroup. All the sequences were clustered and deduplicated via CD-HIT (version 4.8.1) (83) with a 60% identity threshold. The deduplicated sequences were aligned via MAFFT (version 7.505) with the parameters --genafpair --maxiterate 1,000 (84) trimmed with TrimAl (version 1.4.rev15) with the -automated1 parameter (85). A maximum likelihood tree was constructed via IQ-Tree (version 2.0.3) (86) with the model LG + R8 with a bootstrap value of 1,000. The predicted structure of GLUR from the model strain *Nitrosopumilus maritimus* SCM1 was retrieved from the AlphaFold database (AF-A9A551-F1), with an average pLDDT score of 94.25% and 88.05% of residues having a pLDDT score above 90. The structure was then blasted against the PDB database via the Dali server (http://ekhidna2.biocenter.helsinki.fi/dali/) and aligned via the TM-align algorithm (87). The structural alignment results were visualized via PyMOL.

Multiple sequence alignment of 122 archaeal marker genes selected by GTDB-Tk was used to reconstruct the phylogenomic tree of the Nitrososphaeria genome. The representative genome of Nitrososphaeria was selected via dRep (v3.0.0) with the parameters -comp 50 -con 10 -sa 0.95 -nc 0.30 (88). The phylogenomic tree was built via IQ-TREE with the model Q.yeast + F + I + R8 with a bootstrap value of 1,000.

### Statistical analyses

All the statistical analyses were conducted in R (version 4.2.0). The alpha diversity (Shannon index) and beta diversity (NMDS analysis using Bray‒Curtis dissimilarities) of D-AA functional genes were calculated with the ‘vegan’ package on the basis of the relative abundance of genes (TPM). The co-occurrence network was constructed within D-AA functional genes and potential downstream genes, the relevant KOs of which are summarized in Table S3. A prevalence filtering step was applied to remove D-AA functional clusters or KOs of downstream genes that were present in fewer than half of the samples. The relative abundance of genes (TPM) was correlated using Spearman rank correlations, and the *P* values were corrected by the false discovery rate method. Only correlations with an absolute value of Spearman’s correlation coefficient |ρ| >0.7 were retained, which is sufficient to ensure relationships that have *P* < 0.0001.

## Data Availability

The metagenomic data sets generated in this study are available in the National Center for Biotechnology Information repository under accession number PRJNA1169318. The contigs and MAGs were deposited at https://figshare.com/s/71b2b73b73d73146dd7a.

## SUPPLEMENTAL MATERIAL

Supplemental material msystems.00581-25-s0001.docx Fig. S1 to S12; Tables S1 to S3.

Supplemental tables msystems.00581-25-s0002.xlsx Tables S4 to S7.

ASM does not own the copyrights to Supplemental Material that may be linked to, or accessed through, an article. The authors have granted ASM a non-exclusive, world-wide license to publish the Supplemental Material files. Please contact the corresponding author directly for reuse.
