# Supplementary material for: D-amino acid metabolic versatility as a common adaptive strategy in the Mariana Trench microbiome
Source: mSystems. 2025 Jul 11;10(8):e00581-25. doi: 10.1128/msystems.00581-25 (PMC12363236; doi:10.1128/msystems.00581-25)
Supplement: Supplemental material — Fig. S1 to S12; Tables S1 to S3. [file msystems.00581-25-s0001.docx]

**D-amino acid metabolic versatility as a common adaptive strategy in the Mariana trench microbiome**

**Supplementary Figures**

**
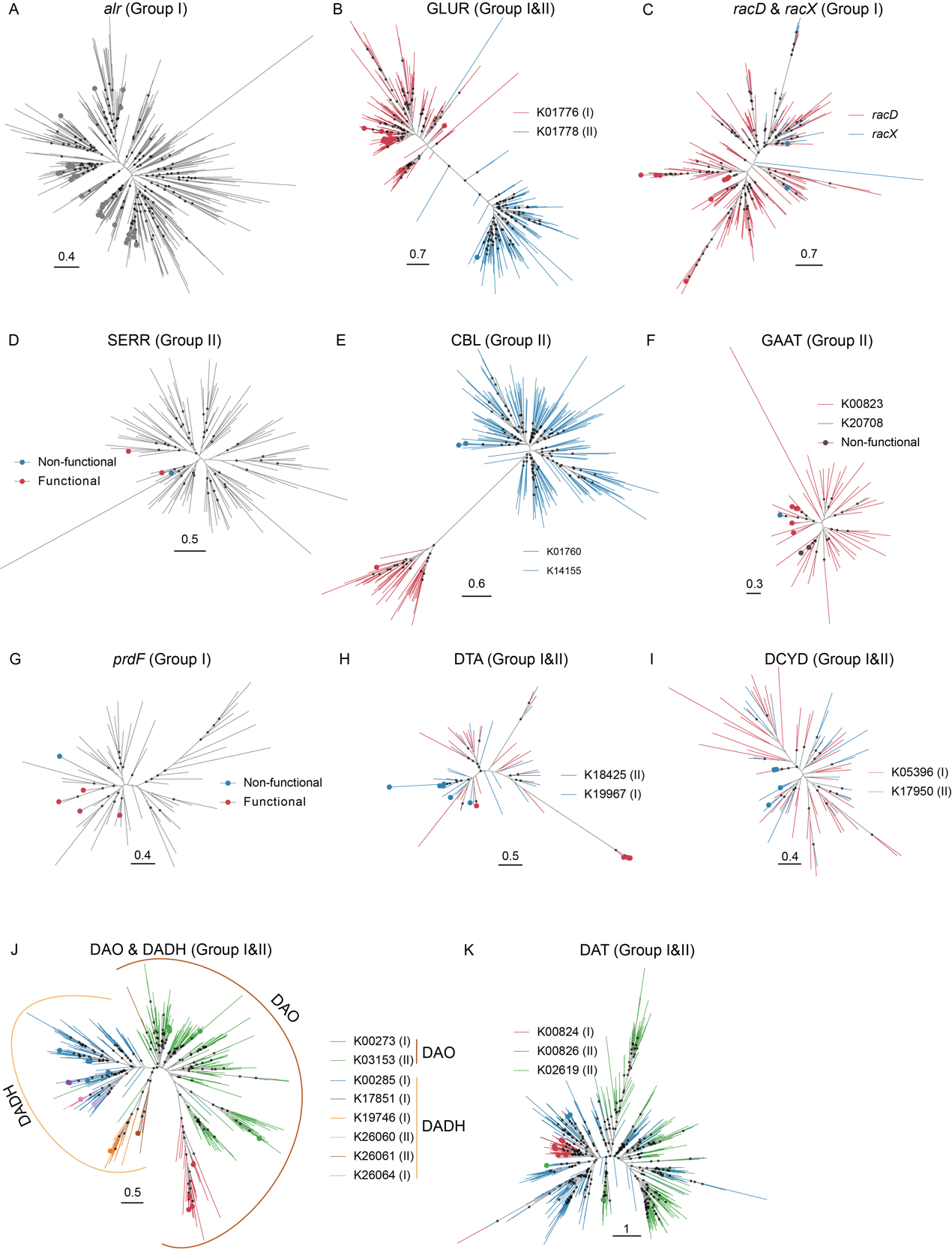
**

**Figure S1** Phylogenetic analysis of Seq-151. The reference sequences were retrieved from the KEGG database and deduplicated via CD-HIT (version 4.8.1) with a 40% identity threshold. The sequences were aligned via MAFFT and trimmed with TrimAl. A maximum likelihood tree was constructed via IQ-Tree with the best model with a bootstrap value of 1,000. Bootstrap values ≥ 90% were shown as black circles on nodes, Seq-151 were shown as circles on tips.

**Figure S2** The relative composition of D-AAs functional clusters across different sample (clusters with relative abundance less than 2% in all of the samples were combined to “other”).


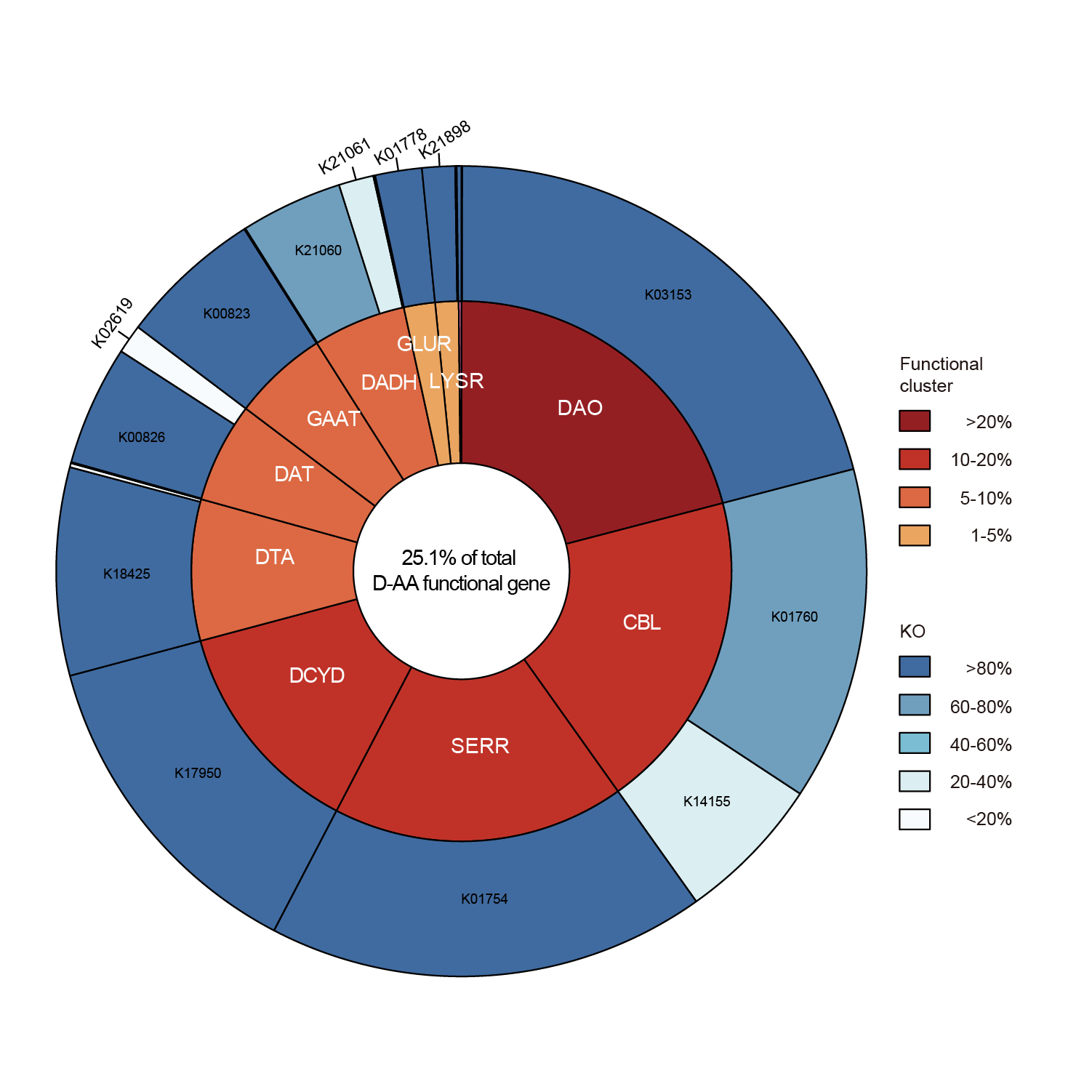


**Figure S3** The composition of D-AAs functional genes annotated supplementally by being aligned with Seq-151.

**Figure S4** The composition of MAGs-mapped D-AAs functional clusters across different sample (clusters with relative abundance less than 2% in all of the samples were combined to “other”).

**Figure S5** The composition of microbial taxa carrying D-AAs functional clusters in MAG-level across different sample (clusters with relative abundance less than 2% in all of the samples were combined to “other”).


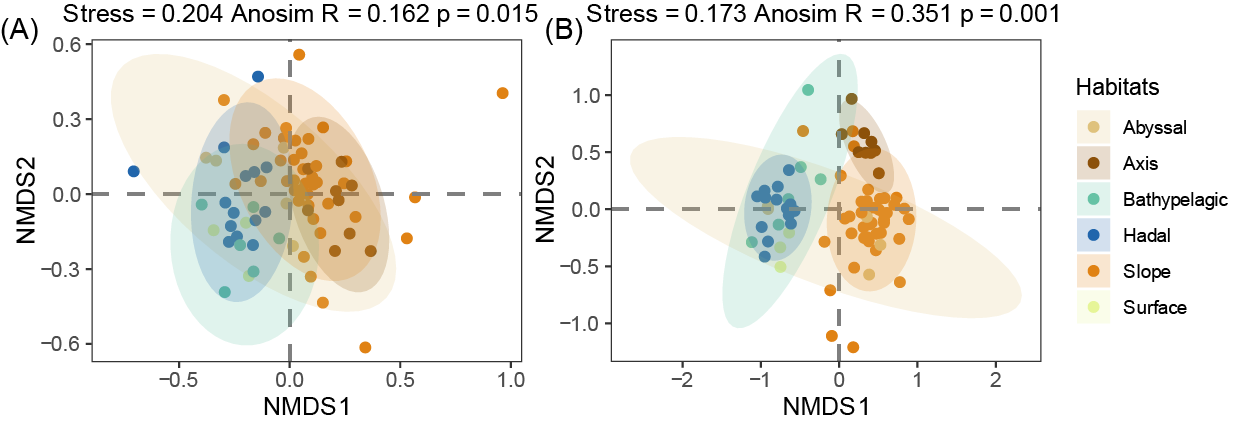


**Figure S6** NMDS analysis of functional clusters (A) and microbial taxa (B, class-level) potentially involved in D-AA metabolism in MAG-level across different habitats. Bray-Curtis dissimilarity was used for distance calculation.

**Figure S7** Distribution of D-AA functional genes in Alphaproteobacteria and Gammaproteobacteria MAGs at order-level. Order belonging to Alphaproteobacteria and Gammaproteobacteria are labeled in orange and blue respectively. The heatmap shows the percentage of MAGs containing different D-AA functional genes across the class level, the number of genomes per class and the number of genomes containing each D-AA functional gene are shown in parentheses, and the number of each D-AA functional gene present in <10% of the genomes is given in squares.


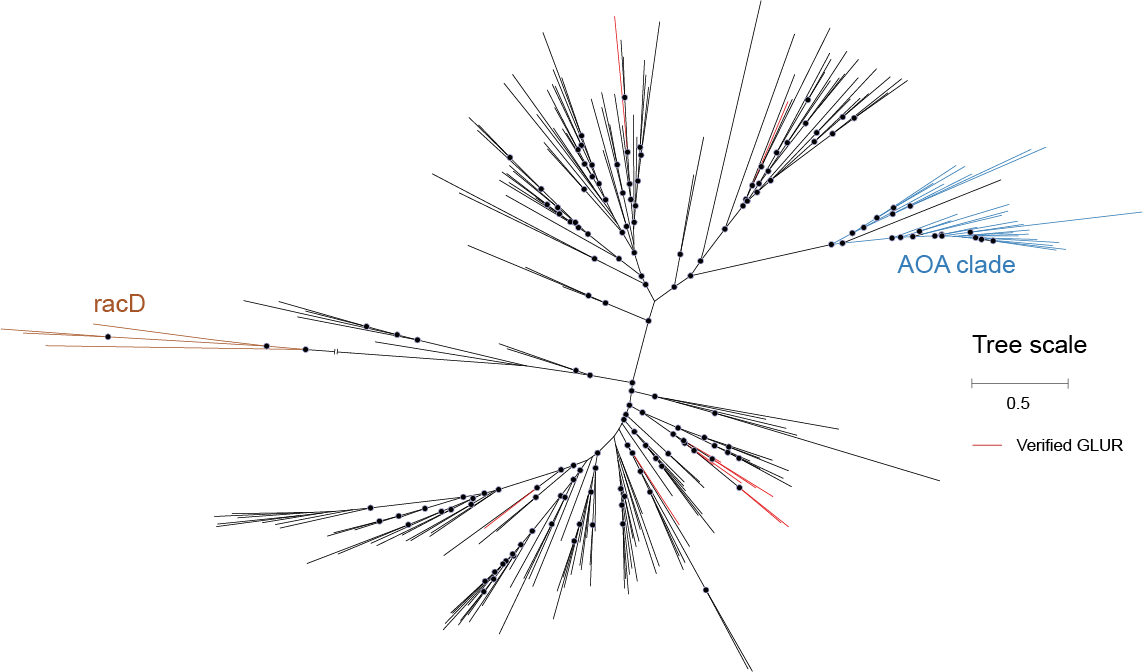


**Figure S8** Maximum likelihood phylogenetic tree of GLUR (K01776). Bootstrap values ≥90% were shown as black circles on nodes, the brown branches were racD, the blue branches were GLUR from AOA, and the rest were the GLUR from bacteria (the red branches were the experimentally verified GLUR).


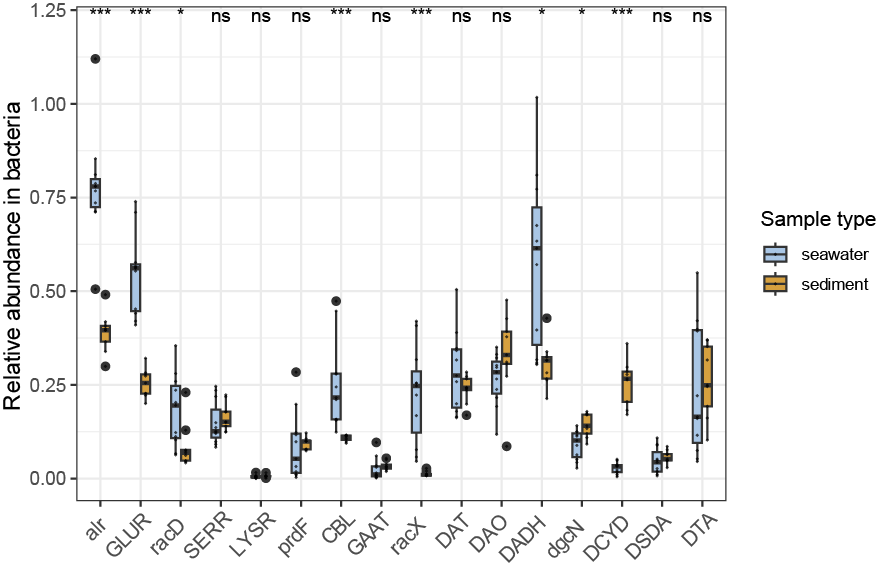


**Figure S9** Comparison of the proportion of bacterial cells containing D-AA functional clusters between seawaters and sediments near the bottom of the Mariana Trench. Statistics were based on the Wilcoxon test. *p < 005; **p < 0.01; ***p < 0.001; ns., no significance.

**Figure S10** Comparison of the relative abundance of MAG-mapped D-AA functional genes among different taxa between seawaters and sediments near the bottom of the Mariana Trench. Order belonging to Alphaproteobacteria and Gammaproteobacteria are labeled in orange and blue respectively.

**Figure S11** (A) Comparison of the relative abundance of MAG-mapped D-AA functional genes among different taxa across seawater samples with different depth. Order belonging to Alphaproteobacteria and Gammaproteobacteria are labeled in orange and blue respectively. (B) Comparison of the relative abundance of MAG-mapped D-AA functional genes among different taxa across sediment samples with different depth.

**Figure S12** Spearman correlation analysis between the proportion of different taxa (Class-level) containing D-AA functional clusters and depth. P values were corrected via the FDR method (*P<0.05; **P<0.01; ***P<0.001).

**Supplementary Tables**

**Table S1** List of 42 D-AA-related KOs available in KEGG database or supplemented from characterized enzymes.

| Functional cluster | KO  category***** | Description of function from KEGG database or comments from published papers |
| --- | --- | --- |
| *alr* | **K01775** | Alanine racemase. |
| GLUR | **K01776** | Glutamate racemase. |
|  | K01778 | Bifunctional diaminopimelate epimerase with glutamate racemase activity. |
| *racD* | **K01779** | Aspartate racemase. |
| SERR | **K12235** | Eukaryotic serine racemase. |
|  | **K18348** | Serine/alanine racemase, vancomycin resistance. |
|  | K01754 | Homologue of eukaryotic serine racemase, bifunctional enzyme with higher catalytic efficiency for racemization than dehydration and strict substrate specificity for serine. |
| *prdF* | **K01777** | Proline racemase. |
| LYSR | **K20707** | Lysine racemase. |
|  | K21898 | Amino acid racemase from hyperthermophile *Thermotoga maritima* with highest substrate speciﬁcity for lysine followed with ornithine. |
|  | K00145 | Lysine racemase from a soil microbial metagenome that can be regarded as a fusion protein of truncated N-acyl amino acid racemase and N-acetyl-γ-glutamyl-phosphate reductase. |
| HISR | **K01780** | Histidine racemase, staphylopine biosynthesis. |
| *bsrV* | **K25317** | PLP-dependent broad-spectrum amino acid racemase. |
| *racX* | **K25316** | PLP-independent broad-spectrum amino acid racemase. |
| CBL | K14155 | Bifunctional enzyme with amino acid racemase activity for multiple amino acids and cystathionine β-lyase activity. |
|  | K01760 | Multifunctional enzyme with broad-spectrum amino acid racemase activity, cystathionine β-lyase activity and serine dehydratase activity. |
| GAAT | K00823 | Broad-spectrum amino acid racemase annotated as γ-aminobutyrate aminotransferase. |
|  | K20708 | Putative γ-aminobutyrate aminotransferase from *Lactobacillus buchneri* catalyzes the racemization of a broad spectrum of nonpolar amino acids. |
| DAO | **K00272** | Eukaryotic D-aspartate oxidase. |
|  | **K00273** | D-amino acid oxidase. |
|  | K03153 | Glycine oxidase with oxidase activity for small D-amino acids such as D-Pro and D-Ala. |
| DADH | **K00285** | D-amino acid dehydrogenase. |
|  | **K19746** | D-arginine dehydrogenase. |
|  | **K17851** | D-proline dehydrogenase. |
|  | **K26064** | D-lysine oxidase. |
|  | K21060 | D-hydroxyproline dehydrogenase with activity towards D-Pro. |
|  | K21061 | D-hydroxyproline dehydrogenase with activity towards D-Pro. |
| *prdA* | **K10793** | D-proline reductase. |
| *dapdh* | K03340 | Bifunctional diaminopimelate dehydrogenase with a relaxed substrate speciﬁcity and potential for D-amino acid synthesis by catalyzing the reductive amination of 2-keto acids. |
| DAT | **K00824** | D-amino acid transaminase. |
|  | K00826 | Putative branched-chain amino acid aminotransferase with aminotransferase activity only for D-AAs but not L-AAs. |
|  | K02619 | D-amino acid transaminase annotated as 4-amino-4-deoxychorismate lyase. |
| *dgcN* | **K26272** | D-glutamate N-acetyltransferase. |
| *aaaT* | K03825 | D-amino acid N-acetyltransferase/succinyltransferase annotated as L-phenylalanine/L-methionine N-acetyltransferase. |
| *cntL* | **K23120** | Histidine 2-aminobutanoyltransferase, staphylopine biosynthesis. |
| *dokD* | **K23385** | D-ornithine/D-lysine decarboxylase. |
| DSDA | **K01753** | D-serine dehydratase. |
|  | **K20498** | Eukaryotic D-serine dehydratase. |
| DCYD | **K05396** | D-cysteine desulfhydrase. |
|  | K17950 | D-cysteine desulfhydrase annotated as L-cysteate sulfo-lyase. |
| DTA | **K19967** | D-threonine aldolase |
|  | K18425 | D-threonine aldolase annotated as 3-hydroxy-D-aspartate aldolase. |

*****KO-25 are highlighted in bold.

**Table S2** Enzymes involved in D-AAs metabolism verified in published papers (Seq-151)

| Organism | Accession* | KO annotation | Substrate (**Product**)** | Ref |
| --- | --- | --- | --- | --- |
| ***alr*** | | | | |
| Pseudomonas aeruginosa PAO1 | AAD47082^a^ | K01775 | Ala, Met, Phe, Arg | [1] |
|  | AAD47081^a^ | K01775 | Ala | [2] |
| Escherichia coli | AAC74274^a^ | K01775 | Ala | [3] |
|  | AAC77023^a^ | K01775 | Ala | [2] |
| Helicobacter pylori NCTC 11637 | BAE93144^a^ | K01775 | Ala, Ser | [4] |
| Clostridium perfringens | ABG82585^a^ | K01775 | Ala | [5] |
| Synechocystis sp. PCC6803 | BAA10510^a^ | K01775 | Ala | [6] |
| Streptococcus iniae | APD32491^a^ | K01775 | Ala, Phe, His | [7] |
| Salmonella enteritidis | CAR35471^a^ | K01775 | Ala | [8] |
|  | CAR35580^a^ | K01775 | Ala | [8] |
|  | CAR32815^a^ | K01775 | Ala | [8] |
| Bacillus anthracis | AAT29333^a^ | K01775 | Ala | [9] |
| Pseudomonas taetrolens | BAH23434^a^ | K01775 | Ala | [10] |
| Pseudomonas putida KT2440 | AAN70834^a^ | K01775 | Ala, Ser, Cys | [11] |
| Vibrio cholerae | AWB72925^a^ | K01775 | Ala, Ser | [12] |
| Streptomyces coelicolor A3 | CAA20401^a^ | K01775 | Ala | [13] |
| Bifidobacterium bifidum | BAC56127^a^ | K01775 | Ala, Ser | [14] |
| Aeromonas hydrophila HBNUAh01 | AGL73884^a^ | K01775 | Ala, Lys, Arg, Val | [15] |
| Bacillus subtilis | NP_389646^a^ | K01775 | Ala | [16] |
| Thermoanaerobacter tengcongensis MB4 | AAM25327^a^ | K01775 | Ala, Arg, Met, Lys, Ser, Val | [17] |
| Bacillus psychrosaccharolyticus | BAA76373^a^ | K01775 | Ala | [18] |
| Bacillus pseudofirmus OF4 | ACE78171^a^ | K01775 | Ala | [19] |
| Lactobacillus sakei ZH-2 | WP_011375306^a^ | K01775 | Ala, Ser, Arg, Pro, Leu, Val | [20] |
| Lactobacillus salivarius UCC118 | Q1WV14^a^ | K01775 | Ala | [21] |
| Corynebacterium glutamicum | Q8RSU9^a^ | K01775 | Ala | [22] |
| Thermoanaerobacter tengcongensis MB4 | AAM24437^a^ | K01775 | Ala, Ser, Arg, Lys, Val | [23] |
| Xanthomonas oryzae | WP_011257590^a^ | K01775 | Ala, Ser | [24] |
| Bacillus licheniformis | WP_011197569^a^ | K01775 | Ala | [25] |
|  | WP_003184708^a^ | K01775 | Ala | [25] |
| Uncultured bacterium | UTQ10504^a^ | K01775 | Ala | [26] |
|  | UTQ10505^a^ | K01775 | Ala | [26] |
| **GLUR** | | | | |
| Thermus thermophilus | BAD71466^a^ | K01776 | Glu | [27] |
| Streptococcus mutans UA159 | AAN59353^a^ | K01776 | Glu | [28] |
| Escherichia coli | P22634^a^ | K01776 | Glu | [29] |
| Helicobacter pylori | Q9ZLT0^a^ | K01776 | Glu | [29] |
| Staphylococcus aureus | Q6GHT5^a^ | K01776 | Glu | [29] |
| Enterococcus faecalis | Q836J0^a^ | K01776 | Glu | [29] |
| Lactobacillus plantarum NC8 | QHM26666^a^ | K01776 | Glu | [30] |
| Acinetobacter baumannii | WP_001063716^a^ | K01776 | Glu | [31] |
| Chlamydia trachomatis | NP_219942^b^ | K01778 | Glu | [32] |
| Bacillus sphaericus ATCC 10208 | AAA68029^a^ | K01776 | Glu | [33] |
| Bacillus anthracis | WP_000774002^a^ | K01776 | Glu | [34] |
| Aquifex pyrophilus | P56868^a^ | K01776 | Glu | [35] |
| Lactobacillus brevis ATCC 8287 | BAA06106^a^ | K01776 | Glu | [36] |
| Pediococcus pentosaceus | AAA16761^a^ | K01776 | Glu | [37] |
| ***prdF*** | | | | |
| Thermococcus litoralis | WP_004067931^a^ | K01777 | Pro | [38] |
| Ferroplasma acidiphilum | AGO60766^a^ | K01777 | Pro | [38] |
| Haloarcula japonica | EMA34308^a^ | K01777 | Pro | [38] |
| Clostridium difficile | Q17ZY4^a^ | K01777 | Pro | [38] |
| Pseudomonas putida KT2440 | AAN66882^c^ | - | Non-functional | [11] |
| ***racD*** | | | | |
| Bifidobacterium bifidum | BAD82810^a^ | K01779 | Asp | [39] |
| Thermococcus litoralis | WP_087036624^a^ | K01779 | Asp | [40] |
| Streptococcus thermophilus | P29079^a^ | K01779 | Asp | [41] |
| Pyrococcus horikoshii | WP_010884762^a^ | K01779 | Asp | [42] |
| DeSulfideococcus Strain SY | BAA12209^a^ | K01779 | Asp | [43] |
| Picrophilus torridus | AAT42734^a^ | K01779 | Asp | [44] |
| Lactobacillus sakei | BAV57431^a^ | K01779 | Asp | [45] |
| Thermococcus sp. KS-8 | BAA35091^a^ | K01779 | Asp | [42] |
| Microcystis aeruginosa PCC7806 | AAF00963^a^ | K01779 | Asp | [46] |
| **SERR** | | | | |
| Enterococcus gallinarum BM4174 | AAD22403^a^ | K18348 | Ser, Ala | [47] |
| Roseobacter litoralis Och 149 | WP_013961474^b^ | K01754 | Ser | [48] |
| Pyrobaculum islandicum | BAE54303^b^ | K01754 | Ser, Thr | [49] |
| Thermotoga maritima | AAD35443^c^ | - | Non-functional | [50] |
| **HISR** | | | | |
| Leuconostoc mesenteroides | BBE07876^b^ | K01775 | His | [51] |
| Staphylococcus aureus | WP_001081693^a^ | K01780 | His | [52] |
| **LYSR** | | | | |
| uncultured bacterium | ACR02674^b^ | K00145 | Lys | [53] |
| Thermotoga maritima | AAD36664^b^ | K21898 | Lys, Arg, Ala, Ser | [54] |
| Oenococcus oeni | ABJ56155^a^ | K20707 | Lys, Arg | [55] |
| Acinetobacter baumannii ATCC 17978 | AKQ27317^b^ | K25317 | Lys | [56] |
| ***racX*** | | | | |
| Bacillus subtilis | P32960^b^ | K01779 | Lys, Arg, His, Ala, Tyr, Phe, Ser, Gln, Met, Asn | [57] |
| Escherichia coli | NP_417317^a^ | K25316 | Met, Leu, Val, His, Asn, Ile, Ala, Ser, Phe | [57] |
| ***bsrV*** | | | | |
| Vibrio cholerae | Q9KSE5^a^ | K25317 | Lys, Arg, Ala, Leu, Met, Ser, Gln, Asn | [12] |
| Pseudomonas putida KT2440 | AAN69319^a^ | K25317 | Lys, Arg, Met, Gln, Ala, Ser, Leu, His, Asn | [11] |
| Pseudomonas taetrolens | BAM13386^a^ | K25317 | Lys, Arg, Met, Leu, Phe | [10] |
| **GAAT** | | | | |
| Pyrococcus horikoshii OT-3 | WP_048053041^b^ | K00823 | Phe, Met, Leu, Ala, Ser, Ile, Val, Trp, Tyr | [58] |
|  | WP_048053213^b^ | K00823 | Ala, Ser, Val | [59] |
|  | WP_048053407^b^ | K00823 | Met, Phe, Leu | [59] |
|  | WP_010885506^c^ | - | Non-functional | [59] |
| Thermococcus litoralis | WP_004069959^b^ | K00823 | Leu, Met | [60] |
| Lactobacillus buchneri | AGE45209^b^ | K20708 | Ile, Val, Leu, Met, Phe, Ser, Ala | [61] |
| Thermococcus kodakarensis | WP_011250162^b^ | K00823 | Leu, Met, Phe, Ala, Ile, Val | [62] |
|  | WP_011251051^c^ | - | Non-functional | [62] |
| **CBL** | | | | |
| Lactobacillus sakei strain LT-13 | BAX66038^b^ | K14155 | Ala, Arg, Asn, Glu, Gln, His, Leu, Lys, Met, Ser, Thr, Trp, Val | [63] |
| Escherichia coli | NP_417481^b^ | K01760 | Ala, Ser, Met, Glu | [64] |
|  | NP_416139^b^ | K14155 | Ala, Ser, Met, Arg, Lys, Phe, Gln, Tyr, Asn | [64] |
| **DADH** | | | | |
| Escherichia coli | P0A6J5^a^ | K00285 | D-Ala, D-Ser, D-Met, D-Phe, D-Pro, D-Trp, D-Val | [65] |
| Rhodothermus marinus JCM9785 | BAR71661^a^ | K00285 | D-Ala, D-Glu, D-Ser, D-Arg, D-Ile, D-Leu, D-His, D-Phe, D-Pro, D-Thr, D-Trp, D-Val, D-Lys | [66] |
| Pyrobaculum islandicum | ABL89010^a^ | K17851 | D-Ala, D-Glu, D-Asp, D-Ser, D-Arg, D-Ile, D-Leu, D-His, D-Phe, D-Pro, D-Thr, D-Trp, D-Val | [67] |
| Sulfideisphaera tokodaii | BAK54626^a^ | K17851 | D-Ala, D-Glu, D-Asp, D-Ser, D-Arg, D-Asn, D-Gln, D-Ile, D-Leu, D-His, D-Phe, D-Pro, D-Thr, D-Trp, D-Tyr, D-Val, D-Lys | [68] |
| Proteus mirabilis JN458 | ARQ85024^a^ | K00285 | D-Ala, D-Ser, D-Asn, D-Met, D-Ile, D-Leu, D-His, D-Phe, D-Pro, D-Thr, D-Trp, D-Tyr, D-Val | [69] |
| Helicobacter pylori NCTC 11637 | BAF48065^a^ | K00285 | D-Ala, D-Ser, D-Phe, D-Pro, D-Val, D-Met, D-Glu, D-Asp | [70] |
| Halomonas sp. LMO_D1 | WP_009286678^a^ | K26064 | D-Ala, D-Asp, D-Ser, D-Arg, D-Asn, D-Gln, D-Met, D-Phe, D-His, D-Val, D-Leu, D-Tyr, D-Trp, D-Thr, D-Ile, D-Arg, D-Lys | [71] |
| Pseudomonas aeruginosa | Q9HXE3^a^ | K19746 | D-Arg, D-Lys, D-Met, D-Phe, D-Tyr, D-His, D-Leu, D-Trp, D-Val, D-Pro, D-Thr, D-Asn, D-Glu, D-Gln, D-Ala | [72] |
|  | Q9HTQ0^a^ | K00285 | D-Ala, D-Arg, D-Asn, D-Gln, D-Met, D-Phe, D-Pro, D-Val | [73] |
|  | PTC34282^a^ | K00285 | D-Glu, D-Pro, D-Gln | [74] |
|  | Q9I477^b^ | K21061 | D-Pro | [75] |
| Pseudomonas putida | Q88NF6^b^ | K21060 | D-Pro | [75] |
| **DAO** | | | | |
| Arthrobacter protophormiae | Q7X2D3^a^ | K00273 | D-Arg, D-Met, D-Leu, D-His, D-Phe, D-Tyr, D-Lys | [76] |
| Rubrobacter xylanophilus | BAP18969^a^ | K00273 | D-Glu, D-Ile, D-Leu, D-His, D-Thr, D-Tyr, D-Val | [77] |
| Uncultured bacterium | AKA66374^b^ | K03153 | D-Arg, D-Met, D-Leu, D-His, D-Phe, D-Tyr, D-Lys | [78] |
| Streptomyces coelicolor | Q9X7P6^a^ | K00273 | D-Val, D-Ile, D-Met, D-Leu, D-Arg, D-Lys | [79] |
| vDAO******* | -^a^ | K00273 | D-Ala, D-Glu, D-His, D-Met | [80] |
| Bacillus subtilis | O31616^b^ | K03153 | D-Ala, D-Val, D-Pro | [81] |
| Geobacillus kaustophilus | Q5L2C2^b^ | K03153 | D-Ala, D-Val, D-Pro, D-Asp, D-Arg, D-Met, D-Phe | [82] |
| Pseudomonas putida | Q88Q83^b^ | K03153 | D-Pro | [83] |
| Bacillus cereus | AAP07738^b^ | K03153 | D-Ala, D-Pro | [84] |
| **Dapdh** | | | | |
| Thermosyntropha lipolytica | MA853431^b^ | K03340 | (**D-Asp, D-Ala, D-Val, D-Glu, D-Leu, D-Ile, D-Met**) | [85] |
| Numidum massiliense | MA853437^b^ | K03340 | (**D-Asp, D-Ala, D-Met, D-Leu, D-Val, D-Ile, D-Glu**) | [86] |
| Symbiobacterium thermophilum | BAD40410^b^ | K03340 | (**D-Ala, D-Leu, D-Val, D-Asp, D-Phe**) | [87] |
| Corynebacterium glutamicum | P04964^c^ | - | Non-functional | [87] |
| ***prdA*** | | | | |
| Clostridium sticklandii | CBH22353^a^ | K10793 | D-Pro | [88] |
| **DAT** | | | | |
| Bacillus sp. strain YM1 | P19938^a^ | K00824 | D-Ala, D-Glu, D-Asp, D-Asn, D-Gln, D-Met, D-Trp (**D-Glu, D-Asp, D-Thr, D-Phe, D-Val**) | [89] |
| Thermotoga maritima | WP_004080810^b^ | K02619 | D-Ala, D-Glu, D-Asp, D-Ser, D-Arg, D-Asn, D-Gln, D-Met, D-Leu, D-Cys, D-Phe, D-Trp, D-Tyr, D-Lys (**D-Glu, D-Asp, D-Ala, D-Thr, D-Leu**) | [90] |
| Aminobacterium colombiense | WP_013049219^b^ | K00826 | D-Glu, D-Ala, D-Asp, D-Leu, D-Phe (**D-Ala, D-Glu**) | [91] |
| Lysinibacillus sphaericus | P54693^a^ | K00824 | D-Ala, D-Glu, D-Asp, D-Arg, D-Asn, D-Gln, D-Met, D-Leu, D-His, D-Phe, D-Trp, D-Lys (**D-Glu, D-Asp, D-Thr, D-Leu, D-Phe, D-Val**) | [92] |
| Parageobacillus toebii SK1 | Q4JFX0^a^ | K00824 | D-Ala, D-Asp, D-Ser, D-Asn, D-His, D-Trp, D-Val (**D-Glu, D-Thr, D-Phe, D-Val**) | [93] |
| Lactobacillus salivarius | Q1WRM6^a^ | K00824 | D-Ala, D-Arg, D-Met, D-Leu, D-His, D-Thr, D-Val (**D-Glu, D-Asp, D-Ala, D-Thr, D-Val, D-Trp, D-Tyr**) | [94] |
| Haliscomenobacter hydrossis | WP_013764869^b^ | K00826 | D-Ala, D-Glu, D-Leu (**D-Glu, D-Ala, D-Thr, D-Phe**) | [95] |
| Mycobacterium smegmatis | AWT56632^b^ | K02619 | D-Ala (**D-Glu**) | [96] |
| Pseudoalteromonas sp. | QQM16034^a^ | K00824 | D-Ala (**D-Glu**) | [97] |
| Oceanicella sp. | QQM16042^a^ | K00824 | D-Ala (**D-Glu**) | [97] |
| Oceanicella sp. | QQM16043^a^ | K00824 | D-Ala (**D-Glu**) | [97] |
| Paraglaciecola polaris | QQM16038^a^ | K00824 | D-Ala (**D-Glu**) | [97] |
| ***dgcN*** | | | | |
| Pseudoalteromonas sp. CF6-2 | USH59575^a^ | K26272 | D-Glu | [98] |
| ***aaaT*** | | | | |
| Geobacillus kaustophilus | BAD75212^b^ | K03825 | D-Arg, D-Lys, D-Val, D-Ala, D-Leu, D-Ile, D-Phe, D-Tyr, D-Trp, D-Ser, D-Thr, D-Asn | [99] |
| ***cntL*** | | | | |
| Staphylococcus aureus | BAB58631^a^ | K23120 | D-His | [52] |
| ***dokD*** | | | | |
| Salmonella enterica | AAL21261^a^ | K23385 | D-Lys | [100] |
| **DTA** | | | | |
| Alcaligenes xylosoxidans | BAA86032^a^ | K19967 | D-Thr | [101] |
| Arthrobacter sp. DK-38 | O82872^a^ | K19967 | D-Thr | [102] |
| Delftia sp. RIT313 | EZP46516^a^ | K19967 | D-Thr | [103] |
| Filomicrobium marinum | CPR22247^a^ | K19967 | D-Thr | [104] |
| Bordetella hinzii | WP_165555284^a^ | K19967 | D-Thr | [104] |
| Afipia carboxidovorans | WP_012563835^a^ | K19967 | D-Thr | [104] |
| Ruegeria pomeroyi | WP_011241927^b^ | K18425 | D-Thr | [105] |
| Pseudomonas aeruginosa | WP_012077937^b^ | K18425 | D-Ala, D-Ser, D-Thr (**D-Thr**) | [106] |
| Pseudomonas protegens | WP_015636124^b^ | K18425 | D-Ala, D-Ser, D-Thr (**D-Thr**) | [106] |
| Singularimonas variicoloris | WP_020650309^b^ | K18425 | D-Thr | [106] |
| Pseudomonas sp. | WP_032861317^b^ | K18425 | D-Ala, D-Ser, D-Thr (**D-Thr**) | [106] |
| **DSDA** | | | | |
| Escherichia coli | P00926^a^ | K01753 | D-Ser, D-Thr | [107] |
| Salmonella typhimurium | Q8ZL08^a^ | K01753 | D-Ser, D-Thr | [108] |
| Pseudomonas aeruginosa | PA3357^a^ | K01753 | D-Ser, D-Thr | [109] |
| Proteus mirabilis | CAR40558^a^ | K01753 | D-Ser | [110] |
| **DCYD** | | | | |
| Escherichia coli | P76316^b^ | K17950 | D-Cys | [111] |
| Salmonella typhimurium | Q8ZNT7^b^ | K17950 | D-Cys, D-Ser | [112] |
| Pectobacterium atrosepticum | Q6D6Z8^b^ | K17950 | D-Cys, D-Ser | [113] |
| Methylorubrum extorquens AM1 | WP_003602931^b^ | K17950 | D-Cys | [114] |

*****Seq-151 was categorized based on their functional characteristics and KO assignment. a. Group I, enzymes exhibiting D-AA-related functions and the annotation was consistent with their function. b. Group II, enzymes exhibiting D-AA-related functions and the annotation was inconsistent with their function. c. Group III, enzymes with high homology to D-AA-related proteins but were experimentally confirmed as nonfunctional.

******For racemases, both D- and L-amino acids are substrates, the prefix of chirality is omitted.

*******Sequence of vDAO that has not been deposited to any database are presented here:

MSQMTRRAFTATGLTAAVAGCATVPPAPGVVMKPVPPVLVSADRVVRVDVGLRPYRASGFRVEREMLGETAVVHNHGHGGGGITLSWGSAQLAVEEGFDPDVAEYAVLGAGALGLSTAMLLLERGAKVTLYAKALSPNTTSNIAGGQWWPASVYDSAAIAPGYMDRHVAAARHSFRRFQLLTGPDYGISWEVNYVLSDRPVTNQPARAGHPMEEFAINVVDYAPGELPFTTAHARSFDTMMVDTPHYLRKLEEDVRERGGRIIVRAFQDAAEVAALDEAVVFNCTGLGAGKLFGDTEIHPVRGQLVILEPQAEIDYNIITGGSAYMFGRRDGIVLGGTFQHHNWSLEPSDADTAAILAANRRLFAGGVEA

**Table S3** List of potential downstream genes of D-AA metabolism.

| Gene | KO | Annotation | Category of metabolism |
| --- | --- | --- | --- |
| LDH | K00016 | L-lactate dehydrogenase | Pyruvate |
| DLAT | K00627 | pyruvate dehydrogenase E2 component | Pyruvate |
| porA | K00169 | pyruvate ferredoxin oxidoreductase alpha subunit | Pyruvate |
| por | K03737 | pyruvate-ferredoxin/flavodoxin oxidoreductase | Pyruvate |
| korA | K00174 | 2-oxoglutarate/2-oxoacid ferredoxin oxidoreductase subunit alpha | Pyruvate/2-keto acid |
| pflD | K00656 | formate C-acetyltransferase | Pyruvate |
| poxB | K00156 | pyruvate dehydrogenase (quinone) | Pyruvate |
| pps | K01007 | pyruvate, water dikinase | Pyruvate |
| PK | K00873 | pyruvate kinase | Pyruvate |
| pyc | K01958 | pyruvate carboxylase | Pyruvate |
| ldhA | K03778 | D-lactate dehydrogenase | Pyruvate |
| ald | K00259 | alanine dehydrogenase | Pyruvate |
| alaA | K14260 | alanine-synthesizing transaminase | Pyruvate |
| ppdK | K01006 | pyruvate, orthophosphate dikinase | Pyruvate |
| pycA | K01959 | pyruvate carboxylase subunit A | Pyruvate |
| bcpA | K01003 | oxaloacetate decarboxylase | 2-keto acid |
| oadA | K01571 | oxaloacetate decarboxylase subunit alpha | 2-keto acid |
| pckA1 | K01596 | phosphoenolpyruvate carboxykinase (GTP) | 2-keto acid |
| pckA2 | K01610 | phosphoenolpyruvate carboxykinase (ATP) | 2-keto acid |
| gudB | K00260 | glutamate dehydrogenase | 2-keto acid |
| gdhA | K00262 | glutamate dehydrogenase (NADP+) | 2-keto acid |
| icd | K00031 | isocitrate dehydrogenase | 2-keto acid |
| mqo | K00116 | malate dehydrogenase (quinone) | 2-keto acid |
| mdh | K00024 | malate dehydrogenase | 2-keto acid |
| IDH3 | K00030 | isocitrate dehydrogenase (NAD+) | 2-keto acid |
| sucA | K00164 | 2-oxoglutarate dehydrogenase E1 component | 2-keto acid |
| kgd | K01616 | multifunctional 2-oxoglutarate metabolism enzyme | 2-keto acid |
| korC | K00177 | 2-oxoglutarate ferredoxin oxidoreductase subunit gamma | 2-keto acid |
| aspB | K00812 | aspartate aminotransferase | 2-keto acid |
| aspC | K00813 | aspartate aminotransferase | 2-keto acid |
| yhdR | K11358 | aspartate aminotransferase | 2-keto acid |
| gltB | K00265 | glutamate synthase (NADPH) large chain | 2-keto acid |
| amoA | K10944 | methane/ammonia monooxygenase subunit A | Ammonia |
| hzs | K20932 | hydrazine synthase subunit | Ammonia |
| glnA | K01915 | glutamine synthetase | Ammonia |
| CPS1 | K01948 | carbamoyl-phosphate synthase (ammonia) | Ammonia |
| ddl | K01921 | D-alanine-D-alanine ligase | Peptidoglycan |
| murD | K01925 | UDP-N-acetylmuramoylalanine--D-glutamate ligase | Peptidoglycan |
| dsrM | K27187 | [DsrC]-trisulfide reductase subunit M | Sulfide |
| asrA | K16950 | anaerobic sulfite reductase subunit A | Sulfide |
| cysJ | K00380 | sulfite reductase (NADPH) flavoprotein alpha-component | Sulfide |
| sir | K00392 | sulfite reductase (ferredoxin) | Sulfide |
| sor | K16952 | Sulfide oxygenase/reductase | Sulfide |
| cysO | K10150 | cysteine synthase | Sulfide |
| cysK | K01738 | cysteine synthase | Sulfide |
| metB | K01739 | cystathionine gamma-synthase | Sulfide |
| metZ | K10764 | O-succinylhomoserine sulfhydrylase | Sulfide |
| fccB | K17229 | sulfide dehydrogenase [flavocytochrome c] flavoprotein chain | Sulfide |

**Table S4.** Information of metagenomic datasets of the Mariana Trench used in this study.

**Table S5.** Relative abundance (TPM) of D-AA functional genes among all clean reads in different samples.

**Table S6.** Relative abundance (TPM) of D-AA functional genes among MAGs in different samples.

**Table S7.** Absolute abundance of functional genes in seawater or sediment samples from different depth. The units of abundance are copies/L for seawater samples and copies/g for sediment samples.

**References**

1. Dong, H., et al., *Enzymatic characterization and crystal structure of biosynthetic alanine racemase from Pseudomonas aeruginosa PAO1.* Biochem Biophys Res Commun, 2018. **503**(4): p. 2319-2325.

2. Strych, U., et al., *Characterization of the alanine racemases from Pseudomonas aeruginosa PAO1.* Curr Microbiol, 2000. **41**(4): p. 290-4.

3. Lambert, M.P. and F.C. Neuhaus, *Mechanism of D-cycloserine action: alanine racemase from Escherichia coli W.* Journal of bacteriology, 1972. **110**(3): p. 978-987.

4. Saito, M., et al., *Alanine racemase from Helicobacter pylori NCTC 11637: purification, characterization and gene cloning.* Life Sci, 2007. **80**(8): p. 788-94.

5. Israr, M., et al., *Biochemical characterization and mutational analysis of alanine racemase from Clostridium perfringens.* J Biosci Bioeng, 2019. **128**(2): p. 149-155.

6. Ashida, H., et al., *Evolution and properties of alanine racemase from Synechocystis sp. PCC6803.* J Biochem, 2022. **171**(4): p. 421-428.

7. Muhammad, M., et al., *Cloning, Biochemical Characterization and Inhibition of Alanine racemase from Streptococcus iniae.* bioRxiv, 2019: p. 611251.

8. Ray, S., et al., *Identification of a new alanine racemase in Salmonella Enteritidis and its contribution to pathogenesis.* Gut pathogens, 2018. **10**: p. 1-17.

9. Couñago, R.M., et al., *Biochemical and structural characterization of alanine racemase from Bacillus anthracis (Ames).* BMC structural biology, 2009. **9**: p. 1-15.

10. Matsui, D., et al., *A periplasmic, pyridoxal-5′-phosphate-dependent amino acid racemase in Pseudomonas taetrolens.* Applied microbiology and biotechnology, 2009. **83**: p. 1045-1054.

11. Radkov, A.D. and L.A. Moe, *Amino acid racemization in Pseudomonas putida KT2440.* J Bacteriol, 2013. **195**(22): p. 5016-24.

12. Espaillat, A., et al., *Structural basis for the broad specificity of a new family of amino-acid racemases.* Acta Crystallogr D Biol Crystallogr, 2014. **70**(Pt 1): p. 79-90.

13. Tassoni, R., et al., *Structural and functional characterization of the alanine racemase from Streptomyces coelicolor A3 (2).* Biochemical and biophysical research communications, 2017. **483**(1): p. 122-128.

14. Yamashita, T., et al., *Molecular characterization of alanine racemase from Bifidobacterium bifidum.* Journal of Molecular Catalysis B: Enzymatic, 2003. **23**(2-6): p. 213-222.

15. Liu, D., et al., *Biochemical characteristics of an alanine racemase from Aeromonas hydrophil HBNUAh01.* Microbiology, 2015. **84**: p. 202-209.

16. Pierce, K.J., S.P. Salifu, and M. Tangney, *Gene cloning and characterization of a second alanine racemase from Bacillus subtilis encoded by yncD.* FEMS microbiology letters, 2008. **283**(1): p. 69-74.

17. Xue, Z., et al., *Characterization and preliminary mutation analysis of a thermostable alanine racemase from Thermoanaerobacter tengcongensis MB4.* Extremophiles, 2013. **17**: p. 611-621.

18. Okubo, Y., et al., *Characterization of Psychrophilic Alanine Racemase fromBacillus psychrosaccharolyticus.* Biochemical and biophysical research communications, 1999. **256**(2): p. 333-340.

19. Ju, J., et al., *Characterization of endogenous pyridoxal 5′-phosphate-dependent alanine racemase from Bacillus pseudofirmus OF4.* Journal of bioscience and bioengineering, 2009. **107**(3): p. 225-229.

20. Kanauchi, M. and N. Matsumoto, *Characteristics of alanine racemase in Lactobacillus sakei ZH‐2 strain.* Food Science & Nutrition, 2023. **11**(8): p. 4745-4755.

21. Kobayashi, J., et al., *Characterization of Lactobacillus salivarius alanine racemase: short-chain carboxylate-activation and the role of A131.* SpringerPlus, 2015. **4**: p. 1-8.

22. Oikawa, T., et al., *Expression of alr gene from Corynebacterium glutamicum ATCC 13032 in Escherichia coli and molecular characterization of the recombinant alanine racemase.* Journal of biotechnology, 2006. **125**(4): p. 503-512.

23. Sun, X., et al., *Crystal structure of a thermostable alanine racemase from Thermoanaerobacter tengcongensis MB4 reveals the role of Gln360 in substrate selection.* Plos one, 2015. **10**(7): p. e0133516.

24. Kang, H.C., et al., *Biochemical Characteristics of an Alanine Racemase from Xanthomonas oryzae pv. oryzae.* Journal of Applied Biological Chemistry, 2011. **54**(4): p. 231-237.

25. Salifu, S.P., K.j. Pierce, and M. Tangney, *Cloning and analysis of two alanine racemase genes from Bacillus licheniformis.* Annals of microbiology, 2008. **58**: p. 287-291.

26. 杨金茹, et al., *Heterologous expression and characterization of alanine racemase derived from fecal microbial metagenomics of Nomascus concolor.* Acta Microbiologica Sinica, 2022. **62**(4): p. 1362-1378.

27. Miyamoto, T., et al., *Enzymatic properties and physiological function of glutamate racemase from Thermus thermophilus.* Biochim Biophys Acta Proteins Proteom, 2020. **1868**(9): p. 140461.

28. Wang, X., et al., *Heterologous expression, purification and biochemical characterization of a glutamate racemase (MurI) from Streptococcus mutans UA159.* PeerJ, 2019. **7**: p. e8300.

29. Lundqvist, T., et al., *Exploitation of structural and regulatory diversity in glutamate racemases.* Nature, 2007. **447**(7146): p. 817-22.

30. Böhmer, N., et al., *Recombinant expression, purification and characterisation of the native glutamate racemase from Lactobacillus plantarum NC8.* Protein expression and purification, 2013. **88**(1): p. 54-60.

31. Hamilton, K.J., *Structural and functional characterisation of glutamate racemase isoform 2 from Acinetobacter baumannii*. 2020, University of Otago.

32. Liechti, G., et al., *Chlamydia trachomatis dapF Encodes a Bifunctional Enzyme Capable of Both d-Glutamate Racemase and Diaminopimelate Epimerase Activities.* mBio, 2018. **9**(2).

33. Fotheringham, I.G., S.A. Bledig, and P.P. Taylor, *Characterization of the genes encoding D-amino acid transaminase and glutamate racemase, two D-glutamate biosynthetic enzymes of Bacillus sphaericus ATCC 10208.* Journal of bacteriology, 1998. **180**(16): p. 4319-4323.

34. Dodd, D., et al., *Functional comparison of the two Bacillus anthracis glutamate racemases.* Journal of bacteriology, 2007. **189**(14): p. 5265-5275.

35. Kim, S.S., et al., *Molecular cloning, expression, and characterization of a thermostable glutamate racemase from a hyperthermophilic bacterium, Aquifex pyrophilus.* Extremophiles, 1999. **3**: p. 175-183.

36. Yagasaki, M., et al., *Cloning, purification, and properties of a cofactor-independent glutamate racemase from Lactobacillus brevis ATCC 8287.* Bioscience, biotechnology, and biochemistry, 1995. **59**(4): p. 610-614.

37. Nakajima, N., et al., *Cloning and expression in Escherichia coli of the glutamate racemase gene from Pediococcus pentosaceus.* Agricultural and biological chemistry, 1986. **50**(11): p. 2823-2830.

38. Watanabe, S., et al., *Identification and Characterization of Bifunctional Proline Racemase/Hydroxyproline Epimerase from Archaea: Discrimination of Substrates and Molecular Evolution.* PLOS ONE, 2015. **10**(3): p. e0120349.

39. Yamashita, T., et al., *Molecular identification of monomeric aspartate racemase from Bifidobacterium bifidum.* Eur J Biochem, 2004. **271**(23-24): p. 4798-803.

40. Washio, T., S. Kato, and T. Oikawa, *Molecular cloning and enzymological characterization of pyridoxal 5'-phosphate independent aspartate racemase from hyperthermophilic archaeon Thermococcus litoralis DSM 5473.* Extremophiles, 2016. **20**(5): p. 711-21.

41. Yohda, M., H. Okada, and H. Kumagai, *Molecular cloning and nucleotide sequencing of the aspartate racemase gene from lactic acid bacteria Streptococcus thermophilus.* Biochimica et Biophysica Acta (BBA)-Gene Structure and Expression, 1991. **1089**(2): p. 234-240.

42. Matsumoto, M., et al., *Occurrence of free D-amino acids and aspartate racemases in hyperthermophilic archaea.* J Bacteriol, 1999. **181**(20): p. 6560-3.

43. Yohda, M., et al., *Gene for aspartate racemase from the Sulfide-dependent hyperthermophilic archaeum, DeSulfideococcus strain SY.* J Biol Chem, 1996. **271**(36): p. 22017-21.

44. Aihara, T., et al., *Structural and functional characterization of aspartate racemase from the acidothermophilic archaeon Picrophilus torridus.* Extremophiles, 2016. **20**(4): p. 385-93.

45. Fujii, T., et al., *Crystallographic studies of aspartate racemase from Lactobacillus sakei NBRC 15893.* Acta Crystallogr F Struct Biol Commun, 2015. **71**(Pt 8): p. 1012-6.

46. Cao, D.D., et al., *Structural insights into the catalysis and substrate specificity of cyanobacterial aspartate racemase McyF.* Biochem Biophys Res Commun, 2019. **514**(4): p. 1108-1114.

47. Arias, C.A., et al., *Serine and alanine racemase activities of VanT: a protein necessary for vancomycin resistance in Enterococcus gallinarum BM4174.* Microbiology (Reading), 2000. **146 ( Pt 7)**: p. 1727-1734.

48. Kubota, T., et al., *Distribution of eukaryotic serine racemases in the bacterial domain and characterization of a representative protein in Roseobacter litoralis Och 149.* Microbiology (Reading), 2016. **162**(1): p. 53-61.

49. Ohnishi, M., et al., *Purification and characterization of serine racemase from a hyperthermophilic archaeon, Pyrobaculum islandicum.* Journal of bacteriology, 2008. **190**(4): p. 1359-1365.

50. Miyamoto, T., et al., *Identification and biochemical characterization of threonine dehydratase from the hyperthermophile Thermotoga maritima.* Amino Acids, 2021. **53**(6): p. 903-915.

51. Adachi, M., et al., *The first identification and characterization of a histidine-specific amino acid racemase, histidine racemase from a lactic acid bacterium, Leuconostoc mesenteroides subsp. sake NBRC 102480.* Amino Acids, 2019. **51**(2): p. 331-343.

52. Ghssein, G., et al., *Biosynthesis of a broad-spectrum nicotianamine-like metallophore in Staphylococcus aureus.* Science, 2016. **352**(6289): p. 1105-9.

53. Chen, I.C., et al., *Isolation and characterization of a novel lysine racemase from a soil metagenomic library.* Appl Environ Microbiol, 2009. **75**(15): p. 5161-6.

54. Miyamoto, T., et al., *Elucidation of the d‐lysine biosynthetic pathway in the hyperthermophile Thermotoga maritima.* The FEBS Journal, 2019. **286**(3): p. 601-614.

55. Kato, S., H. Hemmi, and T. Yoshimura, *Lysine racemase from a lactic acid bacterium, Oenococcus oeni: structural basis of substrate specificity.* J Biochem, 2012. **152**(6): p. 505-8.

56. Le, N.H., et al., *Peptidoglycan editing provides immunity to Acinetobacter baumannii during bacterial warfare.* Sci Adv, 2020. **6**(30): p. eabb5614.

57. Miyamoto, T., et al., *Identification and characterization of novel broad-spectrum amino acid racemases from Escherichia coli and Bacillus subtilis.* Amino Acids, 2017. **49**(11): p. 1885-1894.

58. Kawakami, R., et al., *Identification of a novel amino acid racemase from a hyperthermophilic archaeon Pyrococcus horikoshii OT-3 induced by D-amino acids.* Amino Acids, 2015. **47**(8): p. 1579-87.

59. Kawakami, R., et al., *A Novel PLP-Dependent Alanine/Serine Racemase From the Hyperthermophilic Archaeon Pyrococcus horikoshii OT-3.* Front Microbiol, 2018. **9**: p. 1481.

60. Kawakami, R., et al., *Characterization of a novel moderate-substrate specificity amino acid racemase from the hyperthermophilic archaeon Thermococcus litoralis.* Biosci Biotechnol Biochem, 2021. **85**(7): p. 1650-1657.

61. Mutaguchi, Y., et al., *Identification, purification, and characterization of a novel amino acid racemase, isoleucine 2-epimerase, from Lactobacillus species.* J Bacteriol, 2013. **195**(22): p. 5207-15.

62. Zheng, R.C., et al., *TK1211 Encodes an Amino Acid Racemase towards Leucine and Methionine in the Hyperthermophilic Archaeon Thermococcus kodakarensis.* J Bacteriol, 2021. **203**(7).

63. Kato, S. and T. Oikawa, *A Novel Bifunctional Amino Acid Racemase With Multiple Substrate Specificity, MalY From Lactobacillus sakei LT-13: Genome-Based Identification and Enzymological Characterization.* Front Microbiol, 2018. **9**: p. 403.

64. Miyamoto, T., et al., *Cystathionine beta-lyase is involved in d-amino acid metabolism.* Biochem J, 2018. **475**(8): p. 1397-1410.

65. Olsiewski, P.J., G.J. Kaczorowski, and C. Walsh, *Purification and properties of D-amino acid dehydrogenase, an inducible membrane-bound iron-Sulfide flavoenzyme from Escherichia coli B.* J Biol Chem, 1980. **255**(10): p. 4487-94.

66. Satomura, T., et al., *Dye-linked D-amino acid dehydrogenase from the thermophilic bacterium Rhodothermus marinus JCM9785: characteristics and role in trans-4-hydroxy-L-proline catabolism.* Appl Microbiol Biotechnol, 2015. **99**(10): p. 4265-75.

67. Satomura, T., et al., *Dye-linked D-proline dehydrogenase from hyperthermophilic archaeon Pyrobaculum islandicum is a novel FAD-dependent amino acid dehydrogenase.* J Biol Chem, 2002. **277**(15): p. 12861-7.

68. Satomura, T., et al., *Characterization of dye-linked d-amino acid dehydrogenase from Sulfideisphaera tokodaii expressed using an archaeal recombinant protein expression system.* J Biosci Bioeng, 2020. **130**(3): p. 247-252.

69. Xu, J., et al., *Expression, purification, and characterization of a membrane-bound D-amino acid dehydrogenase from Proteus mirabilis JN458.* Biotechnol Lett, 2017. **39**(10): p. 1559-1566.

70. Tanigawa, M., et al., *D-Amino acid dehydrogenase from Helicobacter pylori NCTC 11637.* Amino Acids, 2010. **38**(1): p. 247-55.

71. Wang, X., et al., *The Capability of Utilizing Abiotic Enantiomers of Amino Acids by Halomonas sp. LMO_D1 Derived From the Mariana Trench.* Frontiers in Astronomy and Space Sciences, 2021. **8**.

72. Li, C., X. Yao, and C.D. Lu, *Regulation of the dauBAR operon and characterization of D-amino acid dehydrogenase DauA in arginine and lysine catabolism of Pseudomonas aeruginosa PAO1.* Microbiology (Reading), 2010. **156**(Pt 1): p. 60-71.

73. He, W., C. Li, and C.D. Lu, *Regulation and characterization of the dadRAX locus for D-amino acid catabolism in Pseudomonas aeruginosa PAO1.* J Bacteriol, 2011. **193**(9): p. 2107-15.

74. He, W., et al., *Functional characterization of the dguRABC locus for D-Glu and d-Gln utilization in Pseudomonas aeruginosa PAO1.* Microbiology (Reading), 2014. **160**(Pt 10): p. 2331-2340.

75. Watanabe, S., et al., *Identification and characterization of D-hydroxyproline dehydrogenase and Δ1-pyrroline-4-hydroxy-2-carboxylate deaminase involved in novel L-hydroxyproline metabolism of bacteria: metabolic convergent evolution.* Journal of Biological Chemistry, 2012. **287**(39): p. 32674-32688.

76. Geueke, B., A. Weckbecker, and W. Hummel, *Overproduction and characterization of a recombinant D-amino acid oxidase from Arthrobacter protophormiae.* Appl Microbiol Biotechnol, 2007. **74**(6): p. 1240-7.

77. Takahashi, S., et al., *A Highly Stable D-Amino Acid Oxidase of the Thermophilic Bacterium Rubrobacter xylanophilus.* Appl Environ Microbiol, 2014. **80**(23): p. 7219-29.

78. Ou, Q., et al., *A novel D-amino acid oxidase from a contaminated agricultural soil metagenome and its characterization.* Antonie Van Leeuwenhoek, 2015. **107**(6): p. 1615-23.

79. Saito, Y., et al., *D-Amino acid oxidase of Streptomyces coelicolor and the effect of D-amino acids on the bacterium.* Annals of Microbiology, 2013. **64**(3): p. 1167-1177.

80. Jian, H., et al., *Diversity and distribution of viruses inhabiting the deepest ocean on Earth.* The ISME Journal, 2021. **15**(10): p. 3094-3110.

81. Nishiya, Y. and T. Imanaka, *Purification and characterization of a novel glycine oxidase fromBacillus subtilis.* FEBS Letters, 1998. **438**(3): p. 263-266.

82. Martinez-Martinez, I., et al., *Characterization and structural modeling of a novel thermostable glycine oxidase from Geobacillus kaustophilus HTA426.* Proteins, 2008. **70**(4): p. 1429-41.

83. EqUAR, M.Y., Y. Tani, and H. Mihara, *Purification and properties of glycine oxidase from pseudomonas putida KT2440.* Journal of nutritional science and vitaminology, 2015. **61**(6): p. 506-510.

84. Seok, J., et al., *Structural basis for stereospecificity to d-amino acid of glycine oxidase from Bacillus cereus ATCC 14579.* Biochem Biophys Res Commun, 2020. **533**(4): p. 824-830.

85. Akita, H., et al., *Characterization of an NAD(P)(+)-dependent meso-diaminopimelate dehydrogenase from Thermosyntropha lipolytica.* Biochim Biophys Acta Proteins Proteom, 2020. **1868**(10): p. 140476.

86. Akita, H., et al., *Identification and functional characterization of NAD(P)(+) -dependent meso-diaminopimelate dehydrogenase from Numidum massiliense.* Microbiologyopen, 2020. **9**(8): p. e1059.

87. Gao, X., et al., *A Newly Determined Member of the meso-Diaminopimelate Dehydrogenase Family with a Broad Substrate Spectrum.* Appl Environ Microbiol, 2017. **83**(11).

88. Kabisch, U.C., et al., *Identification of D-proline reductase from Clostridium sticklandii as a selenoenzyme and indications for a catalytically active pyruvoyl group derived from a cysteine residue by cleavage of a proprotein.* J Biol Chem, 1999. **274**(13): p. 8445-54.

89. Tanizawa, K., et al., *Thermostable D-amino acid aminotransferase from a thermophilic Bacillus species.* Journal of Biological Chemistry, 1989. **264**(5): p. 2445-2449.

90. Miyamoto, T., et al., *Identification of a novel d-amino acid aminotransferase involved in d-glutamate biosynthetic pathways in the hyperthermophile Thermotoga maritima.* FEBS J, 2022.

91. Shilova, S.A., et al., *To the understanding of catalysis by D-amino acid transaminases: a case study of the enzyme from Aminobacterium colombiense.* Molecules, 2023. **28**(5): p. 2109.

92. Yonaha, K., et al., *D-amino acid aminotransferase of Bacillus sphaericus. Enzymologic and spectrometric properties.* Journal of Biological Chemistry, 1975. **250**(17): p. 6983-6989.

93. Lee, S.G., et al., *Functional and structural characterization of thermostable D-amino acid aminotransferases from Geobacillus spp.* Appl Environ Microbiol, 2006. **72**(2): p. 1588-94.

94. Kobayashi, J., et al., *Characterization of d-amino acid aminotransferase from Lactobacillus salivarius.* Journal of Molecular Catalysis B: Enzymatic, 2013. **94**: p. 15-22.

95. Bakunova, A.K., et al., *The Uncommon Active Site of D-Amino Acid Transaminase from Haliscomenobacter hydrossis: Biochemical and Structural Insights into the New Enzyme.* Molecules, 2021. **26**(16).

96. Mortuza, R., et al., *Overexpression of a newly identified d-amino acid transaminase in Mycobacterium smegmatis complements glutamate racemase deletion.* Mol Microbiol, 2018. **107**(2): p. 198-213.

97. Yu, Y., et al., *d-Alanine Metabolism via d-Ala Aminotransferase by a Marine Gammaproteobacterium, Pseudoalteromonas sp. Strain CF6-2.* Appl Environ Microbiol, 2022. **88**(3): p. e0221921.

98. Yu, Y., et al., *Novel D-glutamate catabolic pathway in marine Proteobacteria and halophilic archaea.* ISME J, 2023. **17**(4): p. 537-548.

99. Sakai, A., et al., *Evolution of enzymatic activities in the enolase superfamily: N-succinylamino acid racemase and a new pathway for the irreversible conversion of D- to L-amino acids.* Biochemistry, 2006. **45**(14): p. 4455-62.

100. Phillips, R.S., et al., *STM2360 encodes a d-ornithine/d-lysine decarboxylase in Salmonella enterica serovar typhimurium.* Arch Biochem Biophys, 2017. **634**: p. 83-87.

101. Liu, J., et al., *Gene cloning and overproduction of low-specificity D-threonine aldolase from Alcaligenes xylosoxidans and its application for production of a key intermediate for parkinsonism drug.* Applied microbiology and biotechnology, 2000. **54**: p. 44-51.

102. Kataoka, M., et al., *Isolation and characterization of D-threonine aldolase, a pyridoxal-5'-phosphate-dependent enzyme from Arthrobacter sp. DK-38.* Eur J Biochem, 1997. **248**(2): p. 385-93.

103. Chen, Q., et al., *A new D-threonine aldolase as a promising biocatalyst for highly stereoselective preparation of chiral aromatic β-hydroxy-α-amino acids.* Catalysis Science & Technology, 2017. **7**(24): p. 5964-5973.

104. Park, S.-H., et al., *Cβ-Selective Aldol Addition of d-Threonine Aldolase by Spatial Constraint of Aldehyde Binding.* ACS Catalysis, 2021. **11**(12): p. 6892-6899.

105. Fesko, K., et al., *Four types of threonine aldolases: similarities and differences in kinetics/thermodynamics.* Journal of Molecular Catalysis B: Enzymatic, 2008. **52**: p. 19-26.

106. Fesko, K., G.A. Strohmeier, and R. Breinbauer, *Expanding the threonine aldolase toolbox for the asymmetric synthesis of tertiary α-amino acids.* Applied microbiology and biotechnology, 2015. **99**: p. 9651-9661.

107. Marceau, M., et al., *D-serine dehydratase from Escherichia coli. DNA sequence and identification of catalytically inactive glycine to aspartic acid variants.* Journal of Biological Chemistry, 1988. **263**(32): p. 16926-16933.

108. Bharath, S.R., et al., *Crystal structures of open and closed forms of d‐serine deaminase from Salmonella typhimurium–implications on substrate specificity and catalysis.* The FEBS Journal, 2011. **278**(16): p. 2879-2891.

109. Li, G. and C.D. Lu, *The Cryptic dsdA Gene Encodes a Functional D-Serine Dehydratase in Pseudomonas aeruginosa PAO1.* Curr Microbiol, 2016. **72**(6): p. 788-94.

110. Brauer, A.L., et al., *d-Serine Degradation by Proteus mirabilis Contributes to Fitness during Single-Species and Polymicrobial Catheter-Associated Urinary Tract Infection.* mSphere, 2019. **4**(1).

111. Soutourina, J., S. Blanquet, and P. Plateau, *Role of D-cysteine desulfhydrase in the adaptation of Escherichia coli to D-cysteine.* J Biol Chem, 2001. **276**(44): p. 40864-72.

112. Bharath, S.R., et al., *Structural and mutational studies on substrate specificity and catalysis of Salmonella typhimurium D-cysteine desulfhydrase.* PLoS One, 2012. **7**(5): p. e36267.

113. Xu, X., et al., *Characterization and structural basis of D-cysteine desulfhydrase from Pectobacterium atrosepticum.* Tetrahedron, 2023. **130**: p. 133174.

114. Ekimova, G.A., et al., *Distribution of 1-aminocyclopropane-1-carboxylate deaminase and d-cysteine desulfhydrase genes among type species of the genus Methylobacterium.* Antonie Van Leeuwenhoek, 2018. **111**(10): p. 1723-1734.
